# Putative retina metal/metalloid-binding proteins: molecular functions, biological processes and retina disease associations

**DOI:** 10.1093/mtomcs/mfae045

**Published:** 2024-09-25

**Authors:** Marta Ugarte, Craig Lawless

**Affiliations:** S chool of Medical Sciences, Faculty of Biology, Medicine and Health, University of Manchester, Oxford Road, Manchester M13 9PL, UK; Manchester Royal Eye Hospital, Manchester University NHS Foundation Trust, Oxford Road, Manchester M13 9WL, UK; Wellcome Trust Centre for Cell-Matrix Research, School of Biological Sciences, Faculty of Biology, Medicine and Health Sciences, University of Manchester, Rm A.3034a Michael-Smith Building, Oxford Road, Manchester M13 9PT, UK

## Abstract

The mammalian retina contains high amounts of metals/metalloid-selenium. Their dyshomeostases are associated with certain retinal diseases. We carried out this bioinformatics study to identify the relationships between putative retinal metal/selenium binding proteins, their molecular functions, and biological processes. Identification of putative mouse metal/selenium binding proteins was based on known binding motifs, domains, patterns, and profiles. Annotations were obtained from Uniprot keywords ‘metal binding’, ‘metal ion co-factors’, ‘selenium proteins’. Protein functions were estimated by associative frequency with key words in UniProt annotations. The raw data of five mouse proteomics PRIDE datasets (available to date) were downloaded and processed with Mascot against the mouse taxa of Uniprot (SwissProt/Trembl) and MaxQuant (version 1.6.10.43) for qualitative and quantitative datasets, respectively. Clinically relevant variants were evaluated using archives and aggregated information in ClinVar. The 438 proteins common to all the retina proteomics datasets were used to identify over-represented Gene Ontology categories. The putative mouse retinal metal/metalloid binding proteins identified are mainly involved in: (1) metabolic processes (enzymes), (2) homeostasis, (3) transport (vesicle mediated, transmembrane, along microtubules), (4) cellular localization, (5) regulation of signalling and exocytosis, (6) organelle organization, (7) (de)phosphorylation, and (8) complex assembly. Twenty-one proteins were identified as involved in response to light stimulus and/or visual system development. An association of metal ion binding proteins rhodopsin, photoreceptor specific nuclear receptor, calcium binding protein 4 with disease-related mutations in inherited retinal conditions was identified, where the mutations affected an area within or in close proximity to the metal binding site or domain. These findings suggest a functional role for the putative metal/metalloid binding site in retinal proteins in certain retinal disorders.

## Graphical Abstract

**Graphical Abstract:**
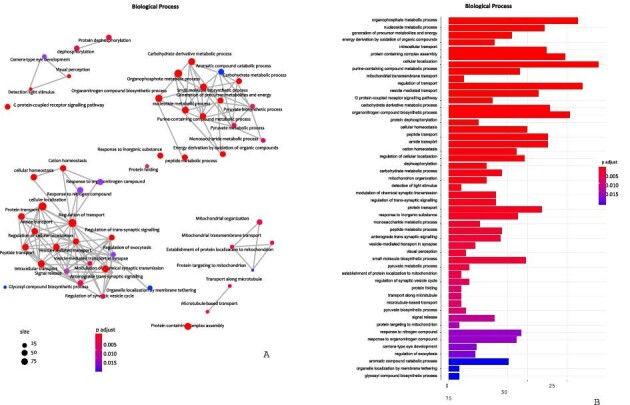
Retinal putative metal/metalloid-binding proteins biological processes.

## Introduction

The biometals zinc, iron, copper, manganese, calcium, magnesium; and the metalloid, selenium, are essential trace elements necessary for normal retinal function.^1–7^ They perform pivotal biochemical functions. Some are key components of metalloproteins/enzymes. They can have an effect on the structure and/or reactivity of enzymes. Other metals/metalloids have specific roles in transport of molecules.

Metalloids have properties intermediate between metals and non-metals. Their physical properties tend to be metallic, whereas their chemical properties tend to be non-metallic. The metalloid, selenium, belongs to the same group as sulphur in the periodic table, and it has unique nucleophilicity, redox potential, ionization, and physicochemical properties.^8^ Selenium combines easily with hydrogen, fluorine, chlorine, and bromine, as well as a number of metals to form compounds called selenides (e.g. magnesium selenide).

The homeostases of metals/metalloid are closely interrelated. What is more, the catalytic activity of various proteins can be supported by more than one metal.

Coordination of metal/metalloids on proteins can play a role in their structure (e.g. folding), biological functions, and/or interactions with other factors. Some metals act as cofactors for proteins to become catalytically active. In multimers, cofactors can be bound either to only one subunit or to the interface. Binding sites are most often a single amino acid residue but, on occasions, a range of residues. In fact, metal ions can be part of well-described metal binding domains such as zinc fingers, EF-hands, and iron sulphur clusters. These binding regions tend to be very conserved in sequence and structure.^9^

Zinc fingers (finger like protrusions) are small, functional, independently folded domains/motifs stabilized by a core of residues (e.g. cysteines, histidines) coordinated to one or more zinc ions (occasionally iron). Cysteine-4 (four cysteine residues) is the most prevalent type of zinc finger, which is found in some nuclear receptors. Zinc finger functions range from DNA-, RNA-, protein- and lipid-binding, to protein-protein interactions, and membrane association. Their binding properties depend on the amino acid sequence of the finger domains, the number of fingers and their structure. Zinc fingers containing proteins play important roles in gene transcription, translation, mRNA trafficking, cytoskeleton organisation, epithelial development, cell adhesion, protein folding, chromatin remodelling, and zinc sensing.^10,11^

The EF-hand motif (helix—loop—helix topology), in turn, is the most common calcium-binding site (Ca²⁺ coordinated by residues in the loop and occasionally Mg^2+^). The functions of proteins that contain EF-hand motifs include calcium buffering in the cytosol and signal transduction.^12^

Specific retinal functions, which might be affected by metal/metalloid-binding proteins include: phototransduction, retinal dark current, phagocytosis of photoreceptor outer segments (OS) by the retinal pigment epithelium (RPE), trans-RPE transport (i.e. transmembrane, vesicle-mediated, along microtubules, endocytosis/exocytosis), the visual cycle and self-renewal of photoreceptor OS. These functions need high amount of energy, substrates, and antioxidant defence; as well as rapid modification of transmembrane transport, the ionic environment, and enzyme activities.^13–16^

Metal metabolic dyshomeostases are thought to be involved in the mechanisms of neurodegenerative disorders such as Alzheimer and Parkinson’s diseases, as well as age-related macular degeneration^17–20^ and diabetic retinopathy.^21^ The metabolic imbalance of metals/metalloid can result in reduced enzymatic activities, elevated protein aggregation, and/or oxidative stress.^22–24^

In order to gain a better understanding of the retinal biological processes where metals/metalloid are involved, we carried out this mouse bioinformatics study. We systematically analysed putative metal/metalloid-binding proteins including metalloproteins (i.e. proteins that require the metal ion to perform their physiological function), as well as proteins involved in metal/metalloid transport, delivery, chaperones, storage, detoxification, and/or efflux. Putative metal/metalloid-binding proteins were identified by the presence of specific metal/metalloid-binding sites and/or domains. We studied their Gene Ontology (GO) annotated molecular functions and biological processes. In addition, we evaluated whether allelic variants in metal binding proteins play a role in known retinal pathophysiological states.

## Methods

### Analysis of putative metal and metalloid-selenium binding proteins

#### Selection of publicly available mouse retina proteomics datasets

Five publicly available proteomic datasets (all measured from normal adult mouse retina) were retrieved from PRoteomics IDEntifications (PRIDE) (https://www.ebi.ac.uk/pride/)^25^ (Table 1). The set of terms used for dataset search was ‘mouse retina proteome’, ‘proteomics’.

**Table 1.** Mouse proteomics datasets found in the PRIDE. The raw data was downloaded and processed with Mascot and MaxQuant for the qualitative and quantitative datasets, respectively

PRIDE accession	Type	Mouse strain	Tissue
PXD009909	Identification/qualitative	Outbred ND4 Swiss Webster	Retinal
PXD009981	Identification/qualitative (phosphorylation enrichment)	Outbred ND4 Swiss Webster	Retinal
PXD003441	Quantitaive (label-free)	Wild-type C57BL/6J	Outer segment & remaining retina
PXD003656	Quantitaive (iTRAQ)	Male C57BL/6	Retina (normal, VEFG & anti-VEGF)
PXD014459	Identification/qualitative	C57BL/6	Retinal

PRIDE is the world's largest data repository of mass spectrometry-based proteomics. It includes protein and peptide identifications (including post-translational modifications), protein and peptide expression values, technical and biological metadata, and any supporting evidence (e.g. peak lists and raw data). PRIDE facilitates the reuse of public proteomics data and disseminates high-quality proteomics evidence into added-value resources, including UniProt^26^ (see below).

The five datasets identified were previously generated with specific primary goals. The main characteristics are summarized below.

**PXD009909:** comprehensive mouse retina proteome. A comprehensive mouse retina proteome using a discovery-based method with retinas from 8 male and 8 female 30-week-old outbred ND4 Swiss Webster mice (three technical repeats for each biological sample). The retinal proteome was identified and subsequently analysed using SEQUEST-HT scoring using reference protein FASTA database from Mus musculus. Only high-scoring peptides, with <1% false discovery rate, were considered for their analysis.

**PXD009981:** comprehensive mouse retina phosphoproteome. These are matched samples to PXD009909, focusing on phosphoproteins via a phosphor-enrichment protocol.

**PXD003441:** mouse retina proteomics. Comprehensive proteomic analysis on light-detecting photoreceptors of OS in the retina, and comparison with the protein profile from the rest of the retina tissue depleted of the OS of 13–15 day old wild-type C57BL/6J mice. Four (24, 25, 26, and 40 retinas) and two biological replicates (30 and 30 retinas) were performed independently in the ‘outer segments’ and the ‘rest of the retina’ experiments, respectively.

**PXD003656:** quantitative proteomics in vascular endothelial growth factor (VEGF)-induced retinal vascular hyperpermeability. Recombinant mouse VEGF164 (100 ng/1.5 μl) (VEGF condition) was injected into the vitreous cavity of the right eye of 6-week-old male C57BL/6 mice. For control samples, phosphate-buffered saline (PBS) (1.5 μl) was injected (control condition). To show the effects of VEGF scavenging, both recombinant mouse VEGF164 (100 ng) and affinity-purified polyclonal antibody against mouse VEGF164 (1 μg) in 1.5 μl of PBS (anti-VEGF condition), were injected. In each of the three conditions (control, VEGF, and anti-VEGF), four samples (six retinas per sample) were obtained. Of the identified peptides, the authors first selected the unique peptides that were detected in two or more of the three experiments.

**PXD014459:** high-pH reversed-phase fractionated neural retina proteome of a normal growing C57BL/6 mouse. Comprehensive wild-type mouse retina proteome prepared using a novel sample preparation approach, the suspension trapping (S-Trap) filter, and further fractionated with high-pH reversed phase chromatography involving a total of 28 injections in the normal C57BL/6 mouse retina.

The raw data of these five mouse proteomics PRIDE datasets were downloaded and processed with Mascot^27^ and MaxQuant (version 1.6.10.43)^28^ for qualitative and quantitative datasets, respectively, against a database, which combined all mouse sequences from Uniprot (SwissProt/Tremble databases) (Supplementary 1).

Mascot is a powerful search engine for identifying proteins and peptides from primary sequence databases by interpreting mass spectrometry data. It compares these molecular weights against a database of known peptides and determines the most likely matches. Mascot then computes a score based on the probability that the peptides from a sample match those in the selected protein database. The more peptides Mascot identifies from a particular protein, the higher the Mascot’s score for that protein. A higher score indicates a more confident match.^27^ Details of the Mascot processing can be seen in Table 2 and full Mascot reports in Supplementary 2 and 3.

**Table 2.** Mascot analysis parameters (PRIDE mouse proteomic datasets PXD009909 and PXD009981)

**Search title**	**PXD009909_PWIZ_normal**
MS data file	70JG.mgf
Database 1	SwissProt 2018_01 (556 568 sequences; 199 530 821 residues)
Database 2	Trembl 2018_01 (107 627 435 sequences; 36 161 263 380 residues)
Taxonomy 1	*Mus musculus* (house mouse) (16 965 sequences)
Taxonomy 2	*Mus musculus* (house mouse) (67 451 sequences)
Timestamp	20 May 2020 at 08:31:20 GMT
Type of search	MS/MS ion search
Quantitation	None
Enzyme	Trypsin
Fixed modifications	Carbamidomethyl (C)
Variable modifications	Acetyl (Protein N-term), Oxidation (M)
Mass values	Monoisotopic
Protein mass	Unrestricted
Peptide mass tolerance	10 ppm
Fragment mass tolerance	0.6 Da
Max missed cleavages	2
Instrument type	ESI-QUAD-TOF
Number of queries	436 445
Significance threshold *P*<	0.05
Max. number of families	AUTO
Ions score or expect cut-off	0
Preferred taxonomy	All entries
Show Percolator scores?	No
**Search title**	**PXD009981_PWIZ_phospho**
MS data file	77JG_A.mgf
Database 1	SwissProt 2018_01 (556 568 sequences; 199 530 821 residues)
Database 2	Trembl 2018_01 (107 627 435 sequences; 36 161 263 380 residues)
Taxonomy 1	*Mus musculus* (house mouse) (16 965 sequences)
Taxonomy 2	*Mus musculus* (house mouse) (67 451 sequences)
Timestamp	22 May 2020 at 04:19:05 GMT
Type of search	MS/MS ion search
Quantitation	None
Enzyme	Trypsin
Fixed modifications	Carbamidomethyl (C)
Variable modifications	Acetyl (Protein N-term),Oxidation (M),Phospho (ST),Phospho (Y)
Mass values	Monoisotopic
Protein mass	Unrestricted
Peptide mass tolerance	10 Da
Fragment mass tolerance	0.6 Da
Max missed cleavages	2
Instrument type	ESI-QUAD-TOF
Number of queries	271 625
Significance threshold *P*<	0.05
Max. number of families	AUTO
Ions score or expect cut-off	0
Preferred taxonomy	All entries
Show percolator scores?	No

MaxQuant software performs mass calibration and database searches for protein identification, using a set of algorithms including peak detection and peptide scoring. It quantifies levels of identified proteins and provides summary statistics by computing ratios of peptides occurring in different samples. The resulting protein ratios are ultimately used to determine quantification intensity profiles. Using the median of peptide ratios, the maximum possible protein quantitation information is achieved.^28^ The MaxQuant parameters matched their counterparts in Mascot, as presented in Table 2.

#### Dataset comparison

The two projects, PXD009909 and PXD009981 were combined, as they both refer to the same biological samples, with the latter being undergoing a phosphor-enrichment sample prep. This resulted in 887 metal/selenium binding proteins representing the two datasets. The four processed datasets were combined to find the overlap of metal binding proteins.

### Visual cycle proteins

A list of 13 visual cycle proteins was obtained from Tsin *et al*.^29^ The PRIDE dataset^25^ was filtered for these proteins with 8 of the 13 being found in all 4 datasets.

### RPE microvilli/interdigitation proteins

A list of 46 RPE microvilli/interdigitation proteins were obtained from Bonilha *et al*.^30^ All non-mouse protein accessions were mapped across to mouse orthologues, resulting in 42 mouse proteins. The list of 42 mouse proteins was used to search the PRIDE datasets,^25^ resulting in 25 proteins present in all proteomic datasets.

### Identification of putative metal/metalloid binding proteins

A list of putative metal/metalloid interacting proteins was acquired by the presence of known specific metal/metalloid-binding motifs, domains, patterns, and profiles. They were estimated by associative frequency with key words in UniProt^26^ annotations^31^ or inferred by sequence homology. We used the following search keywords: ‘metal-binding’, ‘metal ion co-factors’ (KW-0479, https://www.uniprot.org/keywords/KW-0479).

UniProt is a freely accessible collection of databases with information on protein sequences and functions. The central resource, UniProt Knowledgebase (UniProtKB), combines (A) UniProtKB/Swiss-Prot (a reviewed protein set of over 550 000 sequences, where each entry is linked to a summary of experimentally verified, or computationally predicted, functional information created by an expert curator, who organises and summarises it) and (B) UniProtKB/TrEMBL (unreviewed set of a further 60 million sequences largely derived from high throughput DNA sequencing, in which entries are computationally annotated by automated systems).^26^ The integration of these data and the manual curation of protein features, such as functional domains and active sites, amino acid variants, ligand binding sites, and post-translational modifications in the UniProt record, provide mechanistic insights into how, for example, specific variants can lead to disease.

#### Metal binding identification

Metals binding sites and the specific interacting amino acid residues with dedicated annotations in UniProt were identified. Metal binding site annotations on UniProt are based on sequence data or from known or predicted 3D structures from Protein Data Bank (PDB).^32^ The 3D structures shed light on proteins architecture and folding, as well as the interactions with their ligands (e.g. metals/metalloids). They help elucidate binding site residues and the exact position of a residue that causes a genetic disease when it is mutated. Binding annotations most often refer to a single amino acid residue but can sometimes correspond to a range of residues.

The identifiers from the Chemical Entities of Biological Interest (ChEBI) ontology in UniProt describe the nature of the ligand (e.g. CHEBI:33521–metal atom, CHEBI:60240–divalent metal cation). The GO metal ion binding (GO:0046872) in UniProt includes descendants: 2Fe-2S, Iron-sulphur, 3Fe-4S, 4Fe-4S, Haem, LIM domain, Metal-thiolate cluster, zinc-finger.

At present, around 17% of curated proteins from the literature or known structures from PDB have annotated metal binding site residues. In the uncurated TrEMB section, which contains the large majority of known protein sequences, just 3% of proteins have an annotated metal binding site. These annotations are created by a variety of automated annotation methods currently used. The difference between the reviewed (Swiss-Prot) and unreviewed (TrEMBL) suggests there are numerous missing metal binding site annotations in the 225 million TrEMBL sequences at present.

#### GO molecular functions, cellular components and biological process annotations

The GO annotations consider three distinct aspects of how gene product functions can be described: molecular function, cellular component, and biological process.

#### Molecular function

Molecular functions describe activities at the molecular level, such as ‘catalysis’ or ‘transport’. They generally correspond to activities performed by individual gene products (i.e. protein).

#### Biological process

A biological process is the execution of a genetically encoded biological module or program, accomplished by a particular set of molecular functions carried out by specific gene products (or macromolecular complexes), often in a highly regulated manner and in a particular temporal sequence. It consists of all the steps required to achieve the specific biological objective of the module (e.g. detection of light stimulus, DNA repair) (NB, a biological process is not a pathway).

#### Cellular component (location, cellular compartment, structure)

This GO describes locations of gene products in 2 ways: (1) cellular anatomical entities, where the gene product carries out a molecular function (e.g. cytoskeleton, mitochondria, plasma membrane) and (2) stable macromolecular complexes of which they are part (e.g. clathryn complex).

The entire mouse putative metal/metalloid-binding protein list was used as a background from which over-representation was computed.

#### Clinical variants

Clinically relevant variants were evaluated using archive and aggregates information in ClinVar.^33^ This is a freely accessible, public repository of genomic variations and their relationships to human phenotypes, hosted by the National Center for Biotechnology Information (NCBI) and funded by intramural National Institutes of Health (NIH) in the USA, http://www.ncbi.nlm.nih.gov/clinvar. The content of ClinVar includes details of submitter, variation (identified through data collection, clinical testing, research, and literature report) and phenotype, together with interpretation of the relationship among sequence variation, medically important variants and human phenotype, and the evidence supporting each interpretation. The unique combination of submitter, variation, and phenotype determines a record unit and is assigned an accession in the format SCV000000000.0 (SCV).

We obtained a list of clinically relevant variants from ClinVar annotated with the status of pathogenic or likely pathogenic without any conflicting interpretation.

## Results

### Putative metal/metalloid binding proteins identified

#### Whole mouse background

**Fig. 1:**
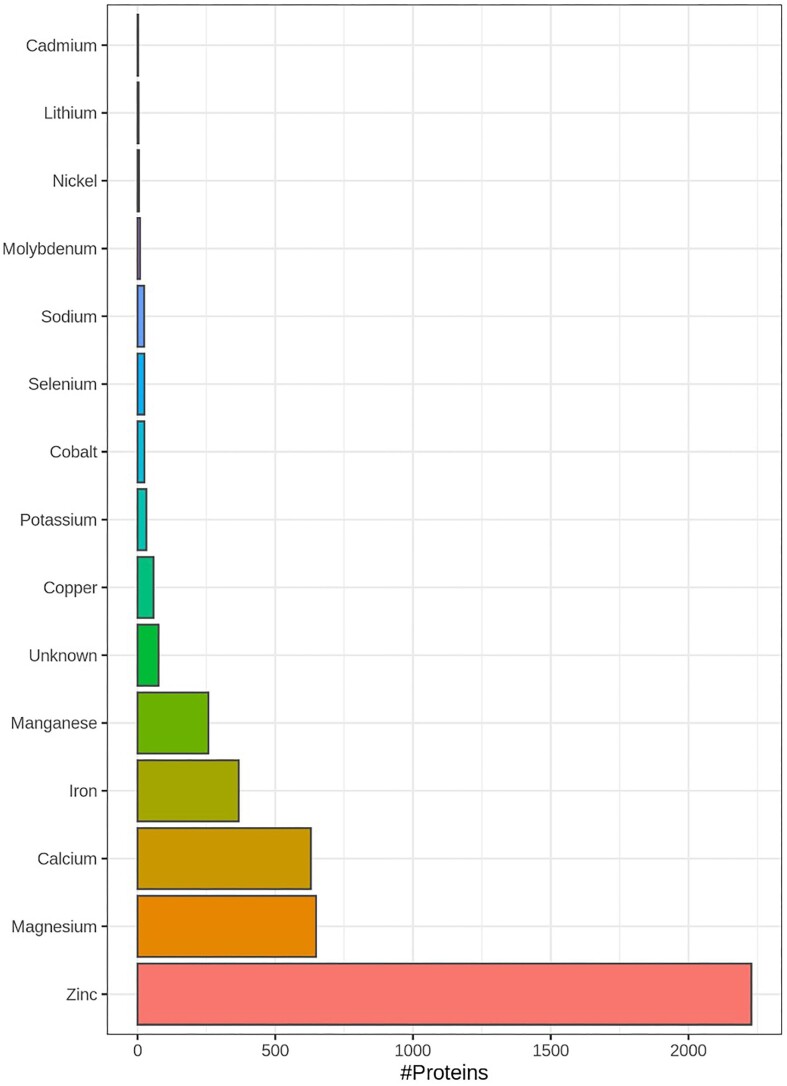
Overview of the number of proteins that can bind Zinc, Magnesium, Calcium, Iron, Manganese, Copper, Potassium, Cobalt, Selenium, Sodium, Molybdenum, Nickel, Lithium, and Cadmium is shown in the histogram. The list of all mouse metal/selenium interacting proteins was obtained from Uniprot, where they were annotated with keyword ‘metal-binding’ (KW-0479, https://www.uniprot.org/keywords/KW-0479), annotated with metal ion co-factors or list as selenium binding proteins in Kryukov et al.^47^ and Guo *et al*.^49^ A total of 3851 proteins were found to be annotated as metal/selenium binding in mouse. The full list can be found in Supplementary 1, with a column listing the metal(s) to which the protein binds. A considerable proportion of proteins can bind more than one metal.

A total of 3851 proteins were found to be annotated as ‘metal/selenium binding’ or ‘metal ion cofactor’ in the whole mouse (Fig. 1). Zinc-binding 2025 proteins (66%), magnesium-binding 457 proteins (12%), calcium-binding 585 proteins (15%), iron-binding 341 proteins (8%), manganese-binding 88 proteins (2%), selenium-binding 25 proteins (0.7%), copper-binding 44 proteins (1%), cobalt-binding 8 proteins (<1%). The full list of proteins can be found in Supplementary 1, with a column listing the metal(s)/metalloid to which the proteins bind to and the metal role. A considerable proportion of proteins can bind more than one metal (Table 3).

**Table 3.** Putative retinal metal/metalloid binding proteins (Mus musculus), binding more than one metal

Protein name gene name (in bold)	Metal/metalloid binding
Acetyl-CoA carboxylase 1 (ACC1) (EC 6.4.1.2) (ACC-alpha) (Acetyl-CoA carboxylase 265) **Acaca**	Magnesium Manganese
Protein argonaute-2 (Argonaute2) (mAgo2) (EC 3.1.26.n2) (Argonaute RISC catalytic component 2) (Eukaryotic translation initiation factor 2C 2) (eIF-2C 2) (eIF2C 2) (Piwi/argonaute family protein meIF2C2) (Protein slicer) **Ago2**	Magnesium Manganese
Manganese-transporting ATPase 13A1 (CATP) (EC 7.2.2.-) **Atp13a1**	Magnesium Manganese
Exosome complex exonuclease RRP44 (EC 3.1.13.-) (EC 3.1.26.-) (Protein DIS3 homolog) (Ribosomal RNA-processing protein 44) **Dis3**	Magnesium Manganese
Endonuclease G, mitochondrial (Endo G) (EC 3.1.30.-) **Endog**	Magnesium Manganese
Isocitrate dehydrogenase [NADP], mitochondrial (IDH) (EC 1.1.1.42) (ICD-M) (IDP) (NADP(+)-specific ICDH) (Oxalosuccinate decarboxylase) **Idh2**	Magnesium Manganese
Isocitrate dehydrogenase [NAD] subunit alpha, mitochondrial (EC 1.1.1.41) (Isocitric dehydrogenase subunit alpha) (NAD(+)-specific ICDH subunit alpha) **Idh3a**	Magnesium Manganese
Isocitrate dehydrogenase [NAD] subunit gamma 1, mitochondrial (Isocitric dehydrogenase subunit gamma) (NAD(+)-specific ICDH subunit gamma) **Idh3g**	Magnesium Manganese
Integrin-linked kinase-associated serine/threonine phosphatase 2C (ILKAP) (EC 3.1.3.16) **Ilkap**	Magnesium Manganese
Inosine triphosphate pyrophosphatase (ITPase) (Inosine triphosphatase) (EC 3.6.1.9) (Non-canonical purine NTP pyrophosphatase) (Non-standard purine NTP pyrophosphatase) (Nucleoside-triphosphate diphosphatase) (Nucleoside-triphosphate pyrophosphatase) (NTPase) **Itpa**	Magnesium Manganese
ATP-dependent RNA helicase SUPV3L1, mitochondrial (EC 3.6.4.13) (Suppressor of var1 3-like protein 1) (SUV3-like protein 1) **Supv3l1**	Magnesium Manganese
Glutamine synthetase (GS) (EC 6.3.1.2) (Glutamate—ammonia ligase) (Palmitoyltransferase GLUL) (EC 2.3.1.225) **Glul**	Magnesium Manganese
ADP-ribose pyrophosphatase, mitochondrial (EC 3.6.1.13) (ADP-ribose diphosphatase) (ADP-ribose phosphohydrolase) (Adenosine diphosphoribose pyrophosphatase) (ADPR-PPase) (Nucleoside diphosphate-linked moiety X motif 9) (Nudix motif 9) **Nudt9**	Magnesium Manganese
Protein phosphatase 1A (EC 3.1.3.16) (Protein phosphatase 2C isoform alpha) (PP2C-alpha) (Protein phosphatase IA) **Ppm1a**	Magnesium Manganese
Protein phosphatase 1B (EC 3.1.3.16) (Protein phosphatase 2C isoform beta) (PP2C-beta) **Ppm1b**	Magnesium Manganese
Protein phosphatase 1E (EC 3.1.3.16) (Ca(2+)/calmodulin- dependent protein kinase phosphatase N) (CaMKP-N) (CaMKP-nucleus) (CaMKN) (Partner of PIX 1) (Partner of PIX-alpha) (Partner of PIXA) **Ppm1e**	Magnesium Manganese
Protein phosphatase 1G (EC 3.1.3.16) (Fibroblast growth factor-inducible protein 13) (FIN13) (Protein phosphatase 1C) (Protein phosphatase 2C isoform gamma) (PP2C-gamma) (Protein phosphatase magnesium-dependent 1 gamma) **Ppm1g**	Magnesium Manganese
Rho-associated protein kinase 2 (EC 2.7.11.1) (Rho-associated, coiled-coil-containing protein kinase 2) (Rho-associated, coiled-coil-containing protein kinase II) (ROCK-II) (p164 ROCK-2) **Rock2**	Magnesium Zinc
Tubulin polymerization-promoting protein (TPPP) (EC 3.6.5.-) (25 kDa brain-specific protein) (TPPP/p25) (p25-alpha) **Tppp**	Magnesium Zinc
CAD protein [Includes: Glutamine-dependent carbamoyl-phosphate synthase (EC 6.3.5.5); Aspartate carbamoyltransferase (EC 2.1.3.2); Dihydroorotase (EC 3.5.2.3)] **Cad**	Magnesium Manganese Zinc
Ethanolaminephosphotransferase 1 (EC 2.7.8.1) (Selenoprotein I) (SelI) **Selenoi**	Magnesium Manganese Selenium
DNA topoisomerase 2-beta (EC 5.6.2.2) (DNA topoisomerase II, beta isozyme) **Top2b**	Magnesium Calcium Manganese
Sarcoplasmic/endoplasmic reticulum calcium ATPase 2 (SERCA2) (SR Ca(2+)-ATPase 2) (EC 7.2.2.10) (Calcium pump 2) (Calcium-transporting ATPase sarcoplasmic reticulum type, slow twitch skeletal muscle isoform) (Endoplasmic reticulum class 1/2 Ca(2+) ATPase) **Atp2a2**	Calcium Magnesium
Ectonucleoside triphosphate diphosphohydrolase 2 (NTPDase 2) (EC 3.6.1.-) (CD39 antigen-like 1) (Ecto-ATP diphosphohydrolase 2) (Ecto-ATPDase 2) (Ecto-ATPase 2) **Entpd2**	Calcium Magnesium
Ectonucleoside triphosphate diphosphohydrolase 5 (NTPDase 5) (EC 3.6.1.6) (CD39 antigen-like 4) (ER-UDPase) (Guanosine-diphosphatase ENTPD5) (GDPase ENTPD5) (EC 3.6.1.42) (Nucleoside diphosphatase) (Uridine-diphosphatase ENTPD5) (UDPase ENTPD5) **Entpd5**	Calcium Magnesium
Plasma membrane calcium-transporting ATPase 1 (EC 7.2.2.10) (Plasma membrane calcium ATPase isoform 1) (PMCA1) (Plasma membrane calcium pump isoform 1) **Atp2b1**	Calcium Magnesium
Plasma membrane calcium-transporting ATPase 4 (PMCA4) (EC 7.2.2.10) **Atp2b4**	Calcium Magnesium
Programmed cell death protein 6 (ALG-257) (Apoptosis-linked gene 2 protein) (ALG-2) (PMP41) **Pdcd6**	Calcium Magnesium
Serine/threonine-protein phosphatase with EF-hands 2 (PPEF-2) (EC 3.1.3.16) **Ppef2**	Calcium Manganese
UDP-glucose:glycoprotein glucosyltransferase 1 (UGT1) (EC 2.4.1.-) (UDP—Glc:glycoprotein glucosyltransferase) (UDP-glucose ceramide glucosyltransferase-like 1) **Uggt1**	Calcium Manganese
Calreticulin (CRP55) (Calregulin) (Endoplasmic reticulum resident protein 60) (ERp60) (HACBP) **Calr**	Calcium Zinc
Dystrophin **Dmd**	Calcium Zinc
Protein piccolo (Aczonin) (Brain-derived HLMN protein) (Multidomain presynaptic cytomatrix protein) **Pclo**	Calcium Zinc
Utrophin **Utrn**	Calcium Zinc
Protein kinase C beta type (PKC-B) (PKC-beta) (EC 2.7.11.13) **Prkcb**	Calcium Zinc
Rabphilin-3A (Exophilin-1) **Rph3a**	Calcium Zinc
Albumin **Alb**	Calcium Copper Zinc
Mitochondrial intermediate peptidase (MIP) (EC 3.4.24.59) **Mipep**	Calcium Cobalt Iron Magnesium Manganese Zinc
4'-phosphopantetheine phosphatase (EC 3.1.3.-) (Inactive pantothenic acid kinase 4) (mPanK4) **Pank4**	Manganese Nickel
Histone lysine demethylase PHF8 (EC 1.14.11.27) (EC 1.14.11.65) (PHD finger protein 8) ([histone H3]-dimethyl-L-lysine(36) demethylase PHF8) ([histone H3]-dimethyl-L-lysine(9) demethylase PHF8) **Phf8-ps**	Iron Zinc
Serine/threonine-protein phosphatase 2B catalytic subunit beta isoform (EC 3.1.3.16) (CAM-PRP catalytic subunit) (Calmodulin-dependent calcineurin A subunit beta isoform) (CNA beta) **Ppp3cb**	Iron Zinc
Serine/threonine-protein phosphatase 2B catalytic subunit gamma isoform (EC 3.1.3.16) (CAM-PRP catalytic subunit) (Calcineurin, testis-specific catalytic subunit) (Calmodulin-dependent calcineurin A subunit gamma isoform) **Ppp3cc**	Iron Zinc
Lysine-specific demethylase 5B (EC 1.14.11.67) (Histone demethylase JARID1B) (Jumonji/ARID domain-containing protein 1B) (PLU-1) ([histone H3]-trimethyl-L-lysine(4) demethylase 5B) **Kdm5b**	Iron Zinc
Alpha-synuclein (Non-A beta component of AD amyloid) (Non-A4 component of amyloid precursor) (NACP) **Snca**	Copper Zinc
Superoxide dismutase [Cu-Zn] (EC 1.15.1.1) **Sod1**	Copper Zinc
Copper chaperone for superoxide dismutase (Superoxide dismutase copper chaperone) **Ccs**	Copper Zinc
Hephaestin (EC 1.-.-.-) **Heph**	Copper Iron
Triokinase/FMN cyclase (Bifunctional ATP-dependent dihydroxyacetone kinase/FAD-AMP lyase (cyclizing)) [Includes: ATP-dependent dihydroxyacetone kinase (DHA kinase) (EC 2.7.1.28) (EC 2.7.1.29) (Glycerone kinase) (Triokinase) (Triose kinase); FAD-AMP lyase (cyclizing) (EC 4.6.1.15) (FAD-AMP lyase (cyclic FMN forming)) (FMN cyclase)] **Tkfc**	Cobalt Magnesium Manganese

### Retinal proteins

**Fig. 2:**
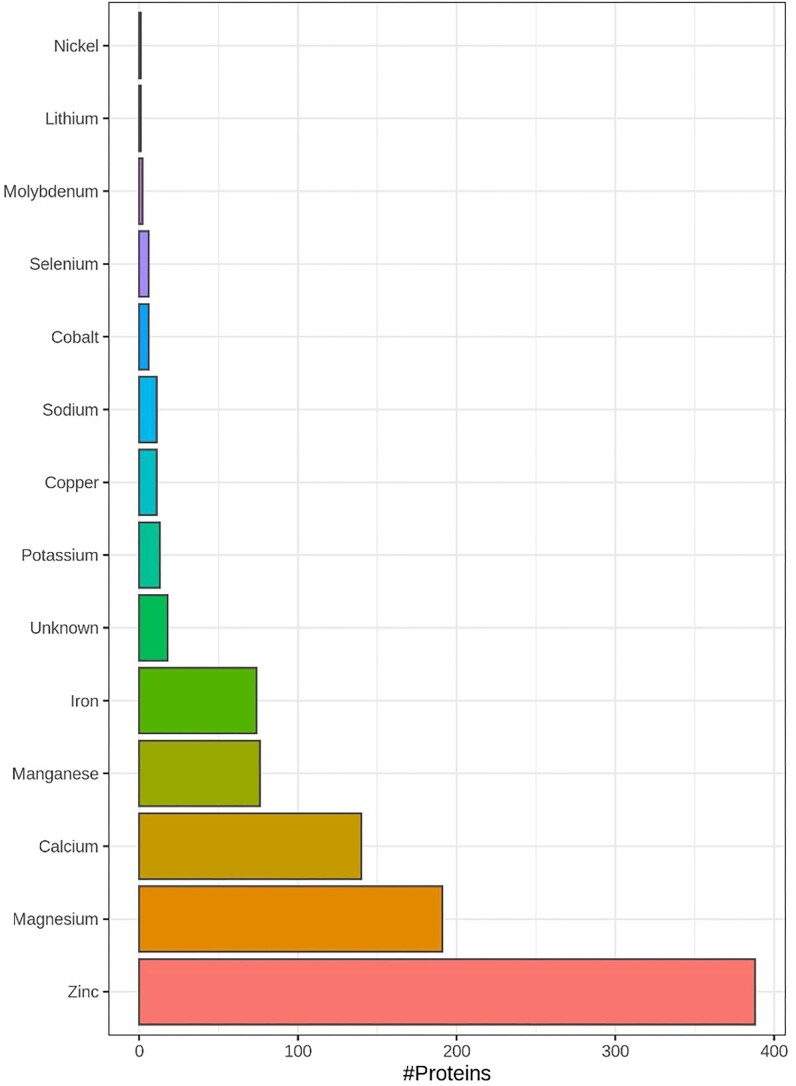
PXD009909: Comprehensive mouse retina proteome. Histogram with number of proteins that can bind Zinc, Magnesium, Calcium, Iron, Manganese, Copper, Potassium, Cobalt, Selenium, Sodium, Molybdenum, Nickel, Lithium by the 799 identified proteins. The raw data were searched using Mascot against the mouse taxa of Uniprot (SwissProt/Trembl) using the identical parameters as detailed in the processing methods of publication. A total of 799 metal/selenium binding proteins were identified at *P* < 0.05 significance threshold, out of a total of ∼5500 proteins families. The full mascot report is in Supplementary 2.

In PXD009909 (comprehensive mouse retina proteome), a total of 799 putative metal/selenium binding proteins were identified at *P* < 0.05 significance threshold, out of a total of ∼5500 protein families (Fig. 2). The full Mascot report can be seen in Supplementary 2.

**Fig. 3:**
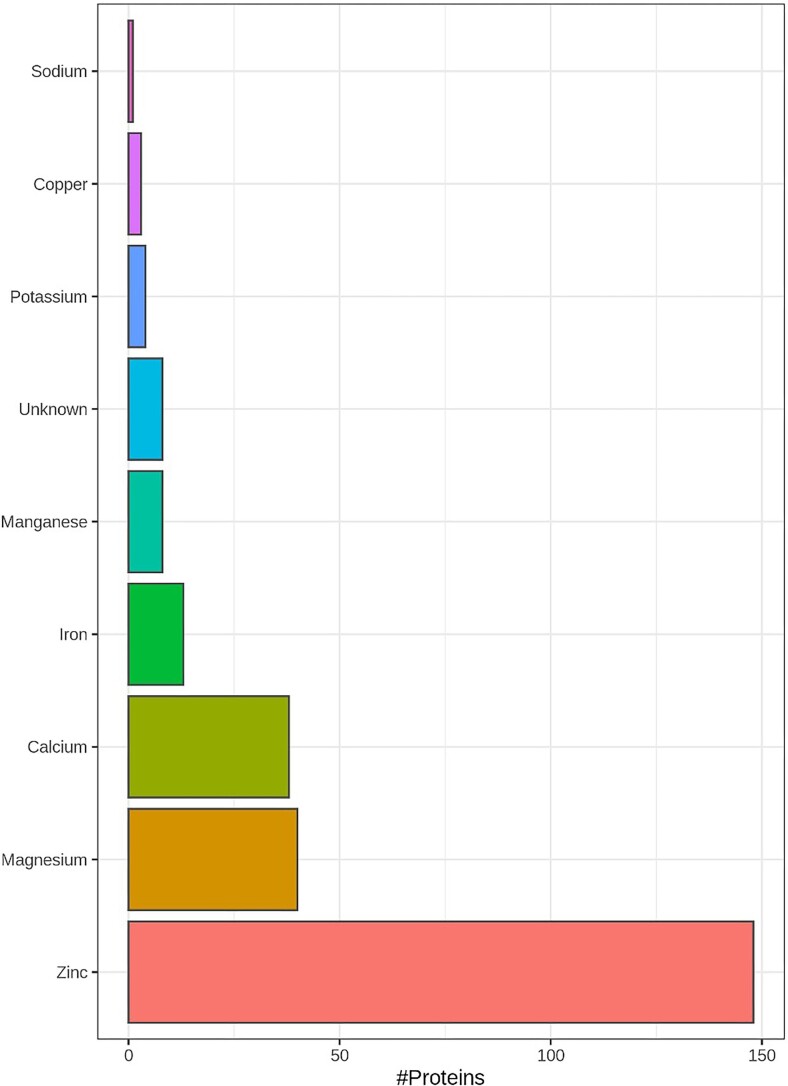
PXD009981: Comprehensive mouse retina phosphoproteome. The histogram shows the number of proteins that can bind Zinc, Magnesium, Calcium, Iron, Manganese, Copper, Potassium, and Sodium by the 299 identified phosphor-proteins. The raw data were searched using Mascot against the mouse taxa of Uniprot (SwissProt/Trembl) using the identical parameters as detailed in the processing methods of publication. These are matched samples to PXD009909, focusing on phosphoproteins via a phosphor-enrichment protocol. A total of 299 metal/selenium binding proteins were identified at *P* < 0.05 significance threshold, out of a total of ∼2000 proteins families. The full mascot report is in Supplementary 3.

In PXD009981 (comprehensive mouse retina phosphoproteome), a total of 228 putative metal/selenium binding proteins were identified at *P* < 0.05 significance threshold, out of a total of ∼2000 protein families (Fig. 3). The full Mascot report in Supplementary 3.

**Fig. 4:**
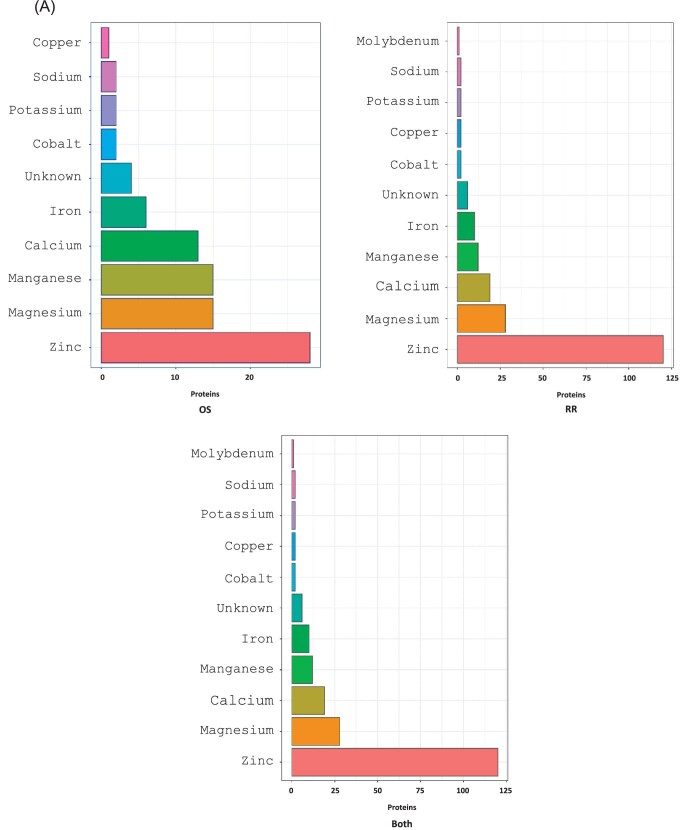

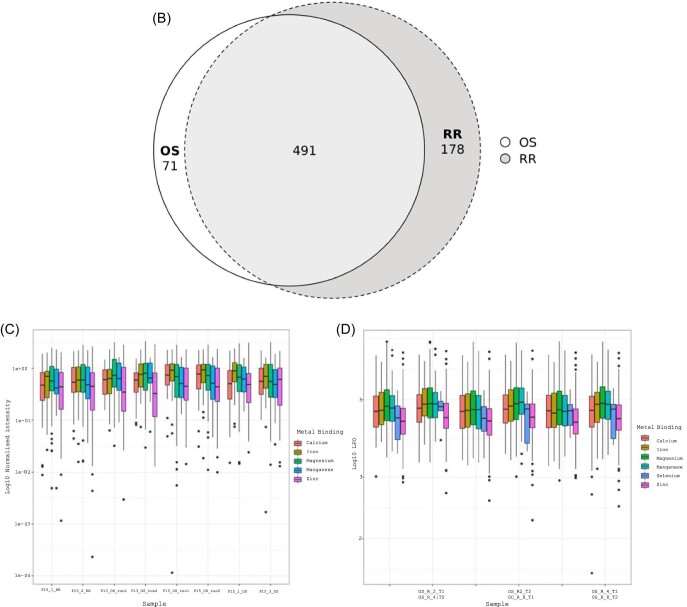
PXD003441**:** Mouse retina proteomics. The three following histograms show the number of proteins that bind Zinc, Magnesium, Calcium, Iron, Manganese, Copper, Potassium, Cobalt, Selenium, Sodium, Molybdenum, Nickel, and Lithium for outer segments (OS), rest of retina (RR) and both. The Venn diagram (B) shows the overlap of metal binding proteins quantified in across the two tissues, OS and RR. The raw data were processed through Maxquant (version 1.6.10.43), following the same search parameters as the publication methods. A total of 814 metal/selenium binding proteins were quantified out of 4424 proteins at a 1% false discovery rate across all samples (i.e. the protein was quantified in at least one sample). (C) The protein intensities were normalised by median-centering to allow sample to sample comparisons. The boxplot shows the distributions of protein abundance (using normalized intensity as a proxy) grouped by the metals (Zinc, Manganese, Magnesium, Iron, Calcium) and Selenium they bind to across, each sample. (D) Boxplot showing the distributions of protein abundance (using LFQ intensity as a proxy) grouped by the metals they bind across each sample.

In PXD003441 (mouse retina proteomics) a total of 814 putative metal/selenium binding proteins were quantified out of 4424 proteins at a 1% false discovery rate across all samples (i.e. the protein was quantified in at least one sample) (Fig. 4). Figure 4B shows the overlap of putative metal/selenium binding proteins quantified across OS and remaining retina (RR). The protein intensities were normalised by median-centring to allow sample to sample comparisons. The boxplots (Fig. 4C, D) show the distributions of protein abundance (using normalised intensity as a proxy) grouped by the metals they bind across each sample. Seventy-eight proteins were identified only in the OS, not the RR.

**Fig. 5:**
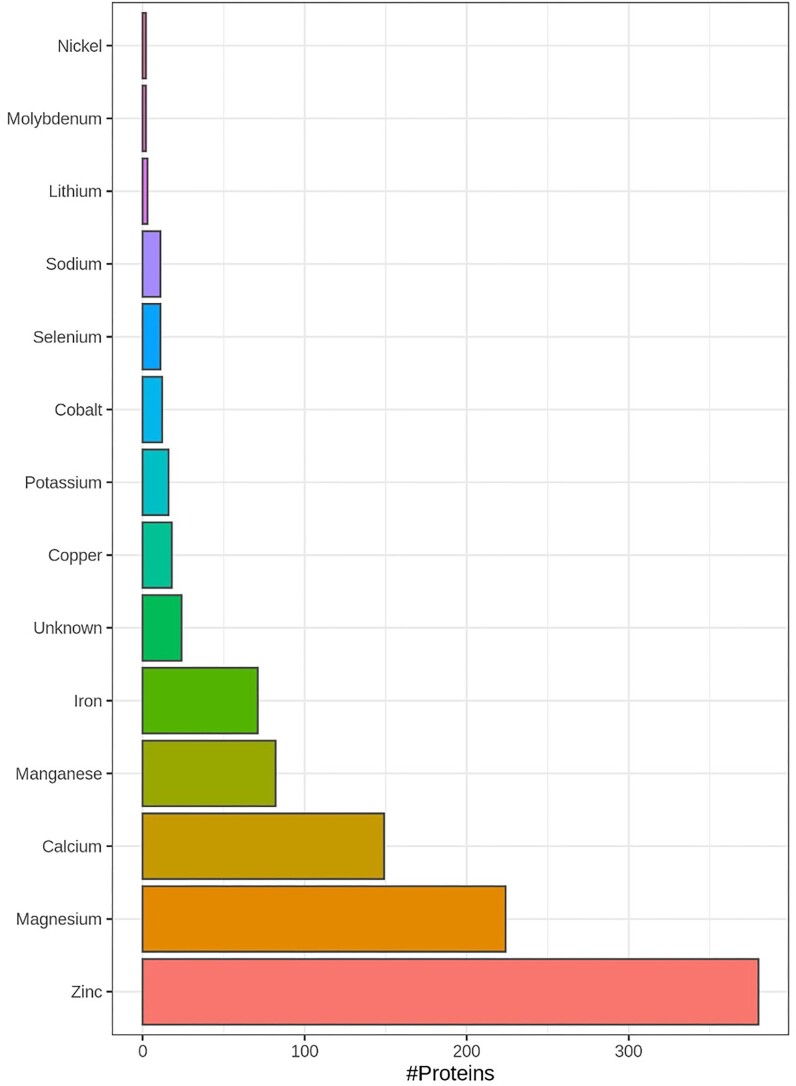
PXD014459: High-pH reversed-phase fractionated neural retina proteome of normal growing C57BL/6 mouse retina proteomics. The histogram shows the number of proteins binding to Zinc, Magnesium, Calcium, Iron, Manganese, Copper, Potassium, Cobalt, Selenium, Sodium, Molybdenum, Nickel, and Lithium, by the 845 identified proteins. The raw data were processed through Maxquant (version 1.6.10.43), following as much as possible the same search parameters as the PRIDE project details. A total of 845 metal/selenium binding proteins were quantified out of 4397 proteins at a 1% false discovery rate across all samples (i.e. the protein was quantified in at least one sample). Using the limited information on the PRIDE project, it appears that there were three biological replicates split into six fractions and repeated as two technical replicates.

In PXD014459 (high-pH reversed-phase fractionated neural retina proteome of normal growing C57BL/6 mouse) (Fig. 5). The raw data were processed through Maxquant (version 1.6.10.43),^28^ following as much as possible the same search parameters as the PRIDE project details.^25^ A total of 845 putative metal/selenium binding proteins were quantified out of 4397 proteins at a 1% false discovery rate across all samples (i.e. the protein was quantified in at least one sample). Using the limited information on the PRIDE project, it appears that there were three biological replicates split into six fractions and repeated as two technical replicates. The histogram (Fig. 5) shows metals bound by the 845 identified proteins. PXD003656 was not included in further analysis.

**Fig. 6:**
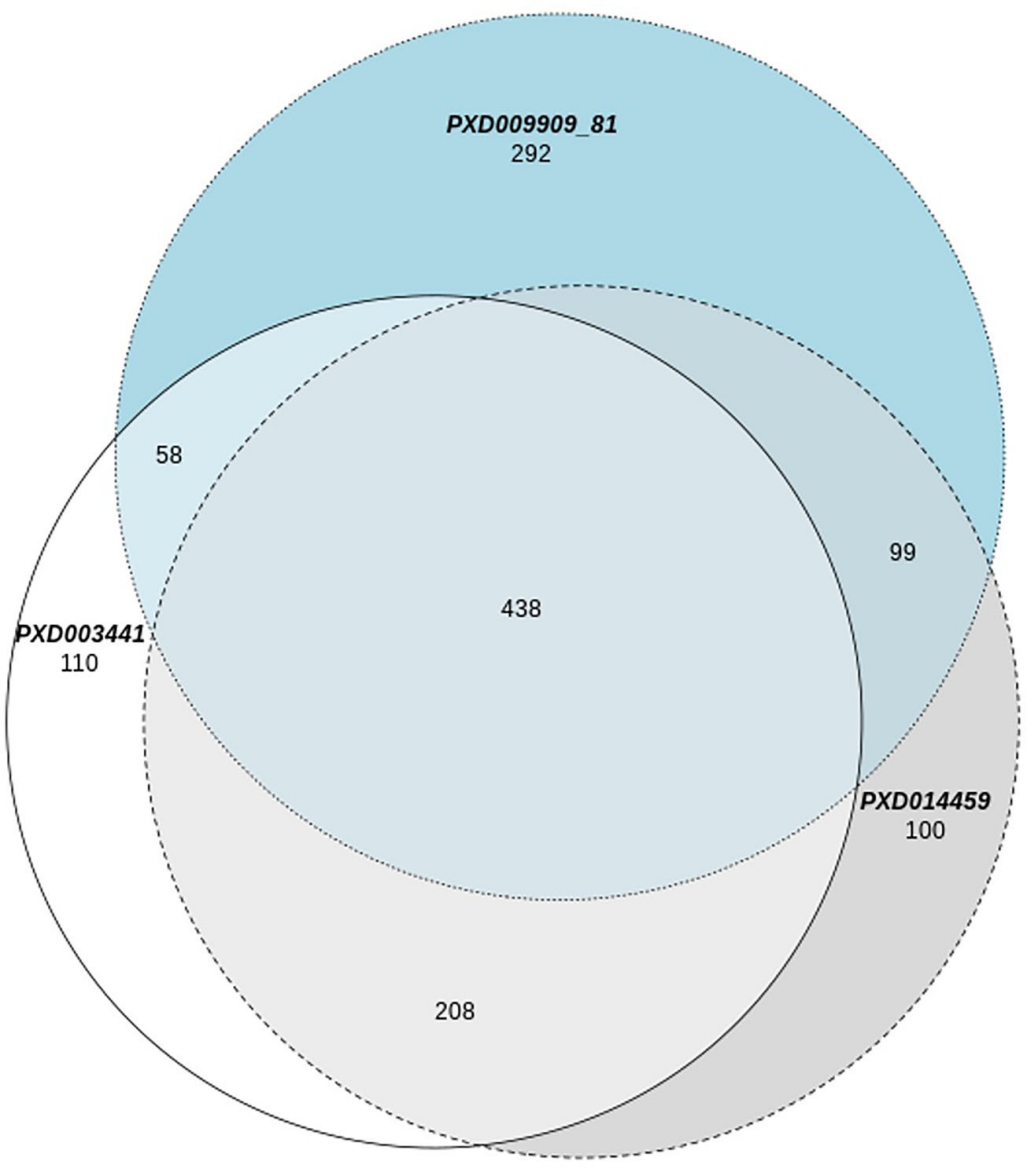
Comparison of the four processed datasets, in order to find the overlap of metal binding proteins. The two projects PXD009909 and PXD009981 were combined (nr) as they both refer to the same biological samples with the latter being undergoing a phosphor-enrichment sample prep. This resulted in 887 metal/selenium binding proteins representing the two datasets. The total number of metal/selenium binding proteins identified in all datasets is 1305, with 438 common to all datasets. The Venn diagram shows the overlap of the 1305 metal binding proteins across the three datasets. The dataset count for each of the metal binding proteins can be found Supplementary 4.

Comparison of the four processed datasets (PXD009909, PXD009981, PXD003441, PXD014459) revealed the total number of putative metal/selenium binding proteins identified in all datasets is 1305, with 438 proteins common to all datasets (Fig. 6). Zinc-binding 121 proteins (31%), magnesium-binding 106 proteins (27%), calcium-binding 88 proteins (22%), iron-binding 33 proteins (8%), manganese-binding 11 proteins (3%), selenium-binding 9 proteins (2.3%), copper-binding 6 proteins (1.5%), cobalt-binding 2 proteins (0.5%). The dataset count for each of the metal binding proteins can be found in Supplementary 4.

**Fig. 7:**
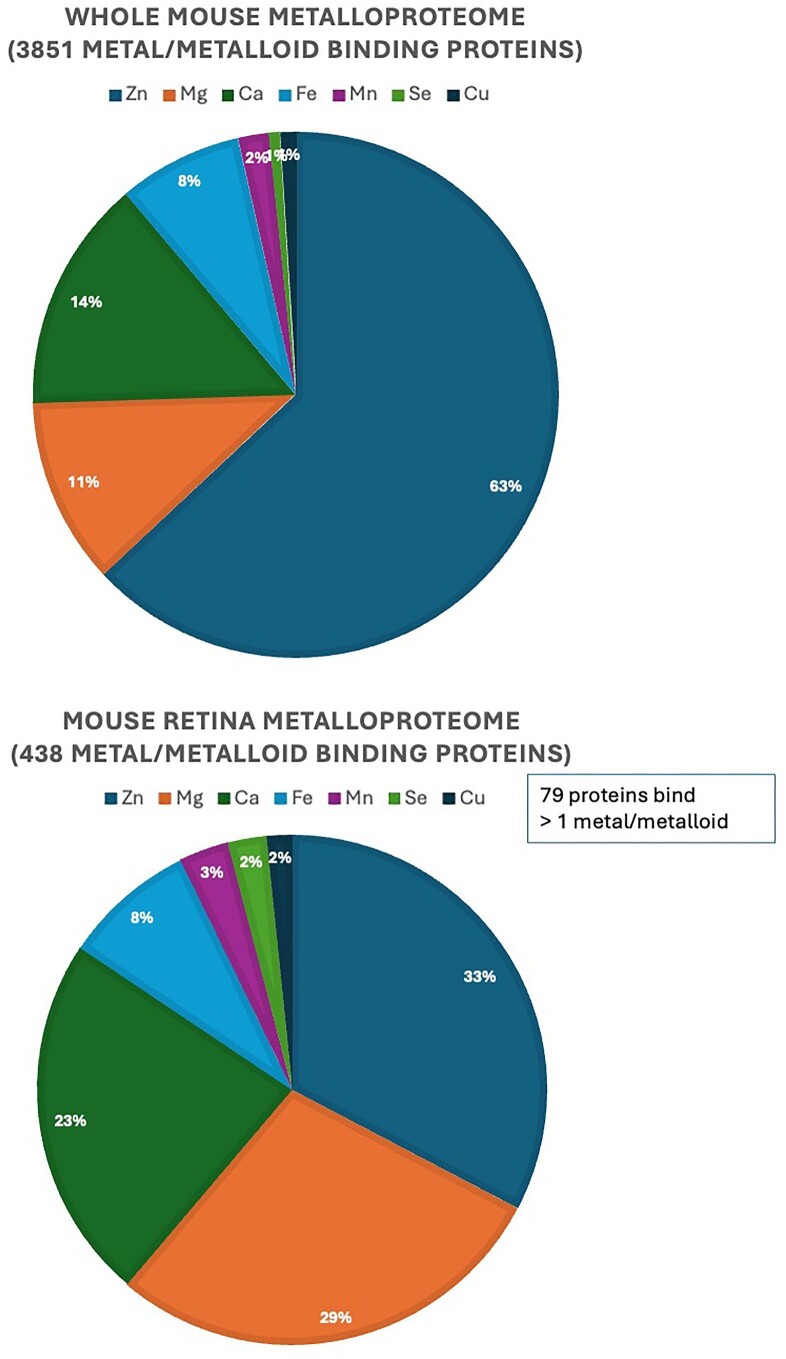
Comparison between the whole mouse and retina tissue, the number of proteins binding Zinc is smaller in the retinal, whereas the number of proteins binding Magnesium and Selenium is higher, supporting the view that Magnesium and Selenium play pivotal roles in retina function.

Comparison of the percentages between the whole mouse and retinal putative metal/metalloid binding proteins suggests there might be a higher proportion of putative magnesium, calcium, and selenium binding proteins in the retina dataset (Fig. 7). Comparing the percentages of putative metal/metalloid binding proteins in the whole mouse vs retinal tissue, we found 12% putative magnesium-binding, 15% putative calcium-binding, 0.7% putative selenium-binding in the whole mouse; whereas the findings in retina tissue revealed 27% putative magnesium-binding, 22% putative calcium-binding, 2.3% putative selenium-binding. These findings suggest high relevance of calcium, magnesium, selenium, their putative dependent and binding proteins, and functions in retinal physiology. Total metal ion concentrations of certain metals in rodent retina have been measured and found to be in high amounts. Magnesium 3458.8 ± 204.9 ug + SEM (5-month-old C57B6 mouse), manganese 2.9 ± 0.6 + SEM (5-month-old C57B6 mouse),^34^ Selenium 0.5 + 0.1 ug/g dry weight (adult rat retina).^35^

Table 3 shows the number of retina proteins annotated to bind specific metals. A significant proportion of proteins bind more than one metal. One hundred and twenty-one putative zinc-binding proteins were identified. Of these 121, 105 (87%) are annotated as binding only zinc. The remaining 16 proteins are annotated as binding calcium, magnesium, iron, copper, and/or manganese, in addition to zinc. One hundred and six proteins are annotated as binding magnesium. Of these 106 proteins, 73 (69%) are annotated as binding only magnesium. Eighty-eight proteins are annotated as binding calcium. Of these 88, 70 (80%) annotated as binding only calcium. Thirty three proteins are annotated as binding iron. Of these 33, 29 (88%) annotated as binding only iron. Eleven proteins annotated as binding manganese. Of these 11, 10 (91%) annotated as binding only manganese. Nine proteins are annotated as binding selenium. Seven of these 9 (78%) binding only selenium. Six proteins annotated as binding copper. Of these 6, 2 (33%) annotated as binding only copper. Two proteins annotated as binding cobalt. One of these two binds only cobalt.

#### Proteins found in RPE microvilli/interdigitation

**Fig. 8:**
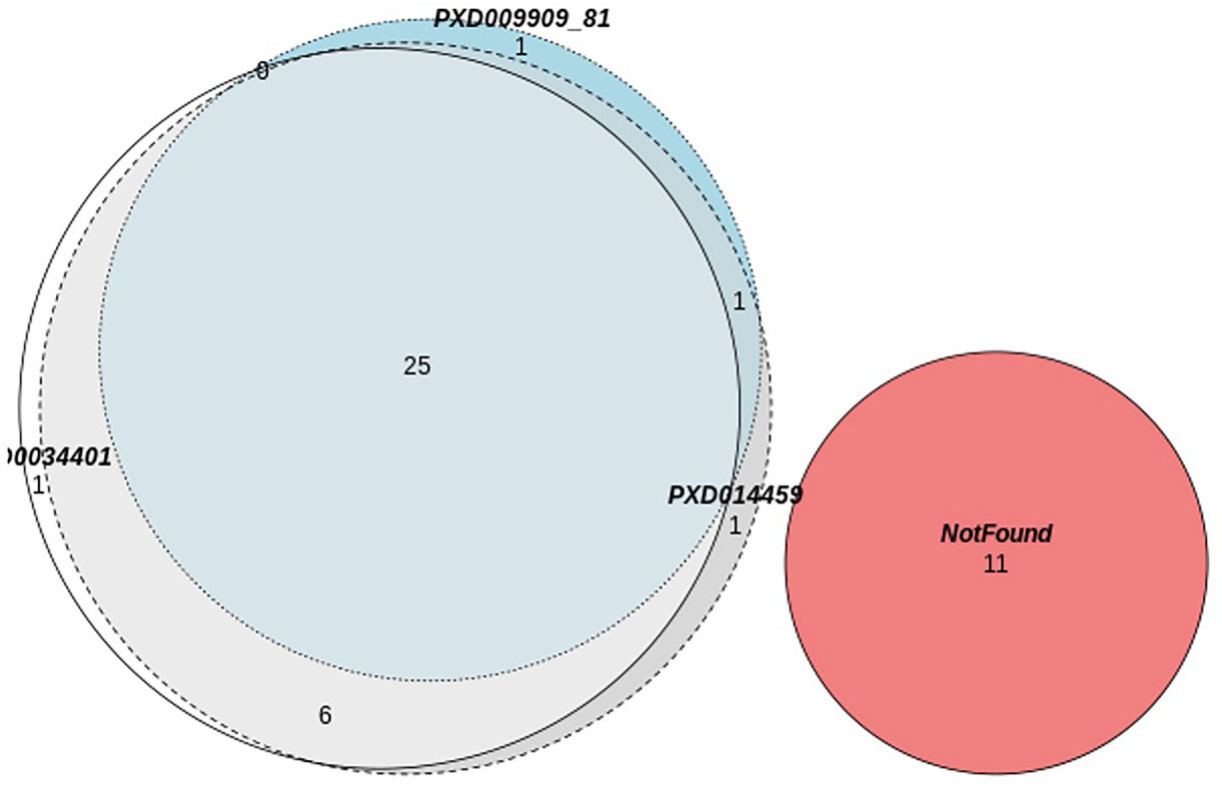
Retinal pigment epithelium microvilli/interdigitation proteins identified in processed datasets. A list of 46 retinal pigment epithelium (RPE) microvilli/interdigitation proteins were obtained from Bonilha *et al*.^30^ All non-mouse protein accessions were mapped across to mouse orthologues, resulting in 42 mouse proteins. The list of 42 mouse proteins was used to search the PRIDE datasets, resulting in 25 proteins present in all proteomic datasets. The overlap of RPE microvilli/interdigitation proteins is shown in the Venn diagram, 11 proteins were not found in any dataset. All protein information for the RPE microvilli/interdigitation can be found in the respective tab in the protein information excel workbook in Supplementary 4.

A list of 46 RPE microvilli/interdigitation proteins was obtained from Bonilha *et al*.^29^ All non-mouse protein accessions were mapped across to mouse orthologues, resulting in 42 mouse proteins, which were used to search the PRIDE datasets (PXD009909, PXD009981, and PXD003441). Twenty-five proteins were present in all proteomic datasets (Fig. 8). The overlap of RPE microvilli/interdigitation proteins is shown in the Venn diagram, 11 proteins were not found in any dataset. All protein information for the RPE microvilli/interdigitation can be found in Supplementary 5.

#### GO molecular functions, cellular components, and biological processes where putative retinal metal-binding proteins are involved

**Fig. 9:**
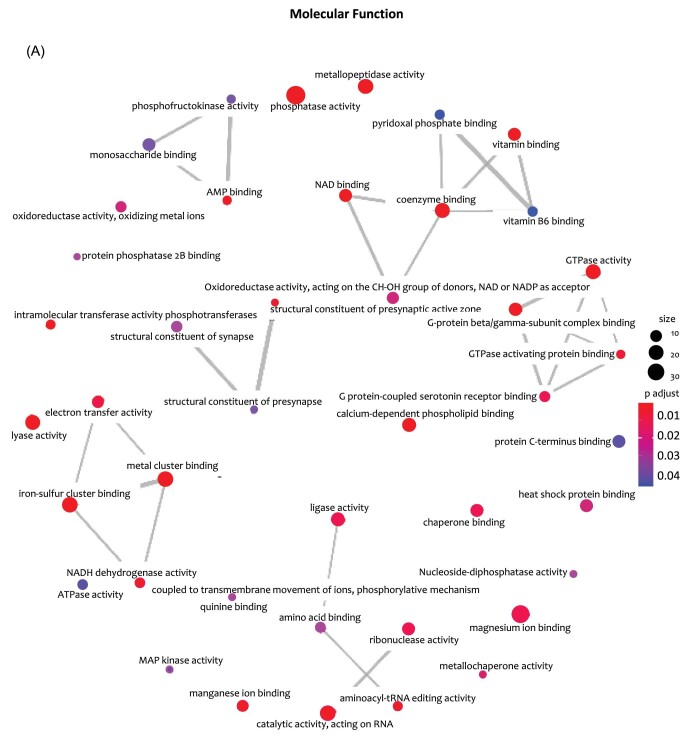

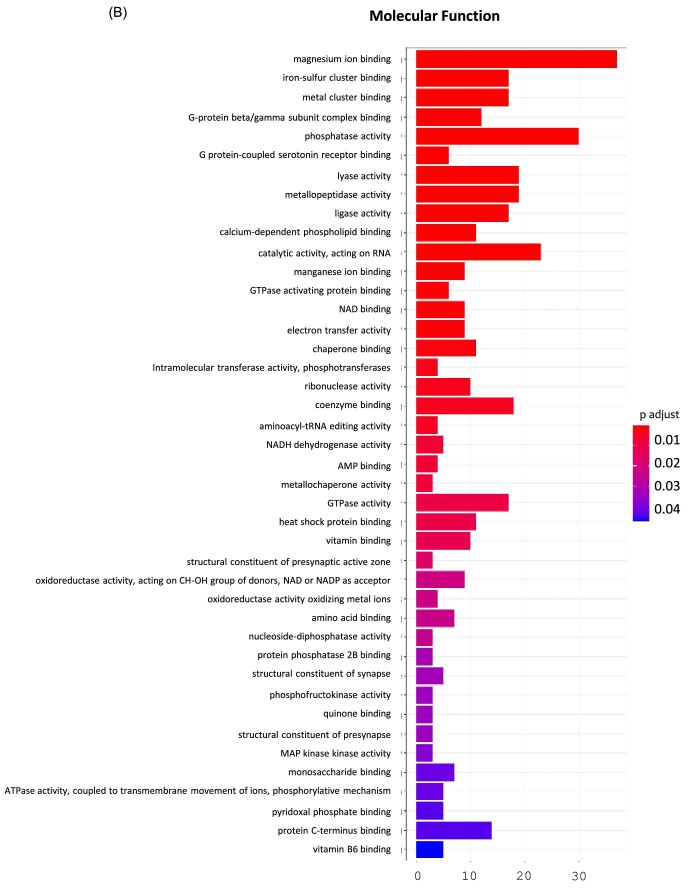
The networks below show the protein-connections of the 26 molecular function categories (A) adjusted *P*-value of 0.05. (B) The 438 proteins common to all datasets was used to identify over-represented gene ontology (GO) categories. The entire mouse metalloproteome was used as a background from which to compute over-representation. The full GO over-representation results can be found in Supplementary 5 (425 of 438) The barplots show all 26 molecular function categories (A) adjusted *P*-value of 0.05 (A) adjusted *P*-value of 0.05 and top 30 (50 of 73?) over-represented GO categories for biological process (B). The networks show the protein-connections of the top 30 over-represented GO categories for biological process (A) below adjusted *P*-value of 0.05 and the 26 molecular function categories below adjusted *P*-value of 0.05. (B) The 438 proteins common to all datasets was used to identify over-represented GO categories. The entire mouse metalloproteome was used as a background from which to compute over-representation. The full GO over-representation results can be found in Supplementary 5 (425 of 438). The barplots show all 26 molecular function categories (A) adjusted *P*-value of 0.05 (A) adjusted *P*-value of 0.05 and top 30 (50 of 73?) over-represented GO categories for biological process (B).

Using the 438 retinal putative metal/metalloid-binding proteins common to all processed datasets, we identified over-represented GO categories for biological process (30) (A) adjusted *P*-value of 0.05 and the 26 molecular function categories (B) adjusted *P*-value of 0.05 can be seen in Fig. 9. The whole mouse putative metal binding proteins dataset (Fig. 1 and Supplementary 1) was used as background for evaluation of over-representation. The full GO over-representation results can be found in Supplementary 4.

#### Molecular functions

Over-represented GO Molecular Functions of retinal putative metal/metalloid-binding proteins found (Fig. 9) binding to diverse molecules and activities such as phosphatase, lyase, peptidase.

Binding to coenzyme, iron sulphur cluster, protein C terminus, protein beta/gamma subunit, calcium-dependent phospholipid, heat shock protein, chaperone, vitamin, NAD, amino acid, monosaccharide, GTPase activating protein, G-protein coupled, serotonin, AMP, quinone, protein phosphatase 2B, pyridoxal phosphate.

Over-represented enzyme activities include: phosphatase, catalysis on RNA, lyase, metallopeptidase, ligase, GPTase, ribonuclease, electron transfer, oxidoreductase, ATPase, NADH dehydrogenase, phosphotransferase, aminoacyl-tRNA editing, metallochaperone, nucleoside-diphosphatase, phosphofructokinase, MAP kinase.

### Cellular components of retinal metal/metalloid-binding proteins

Cellular anatomical entities, where retinal metal/metalloid-binding proteins carry out their molecular function include: (1) mitochondrion (envelope, membrane, protein complex), (2) myelin sheath, (3) plasma membrane bound cell projections, and (4) neuron projections.

### GO biological processes of retinal metal/metalloid binding proteins

**Fig. 10:**
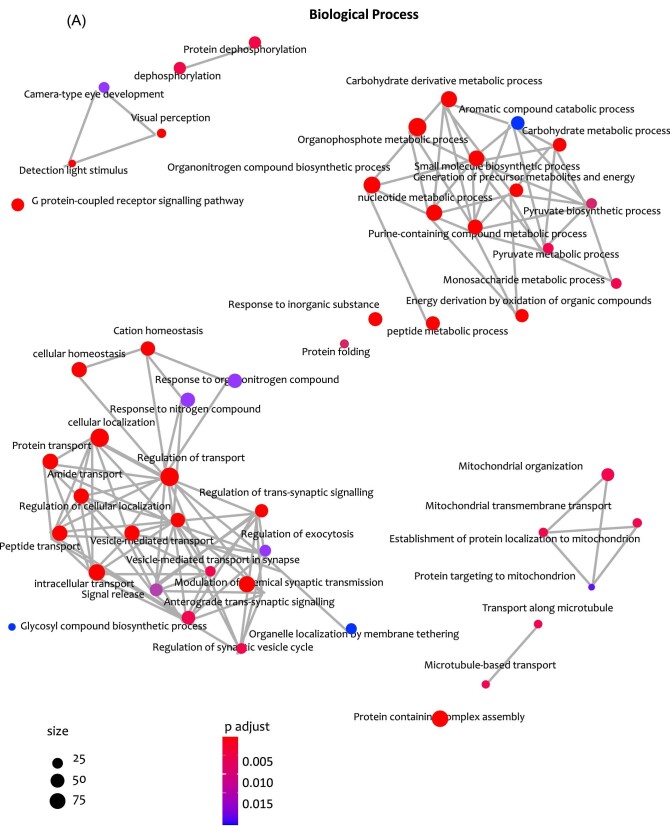

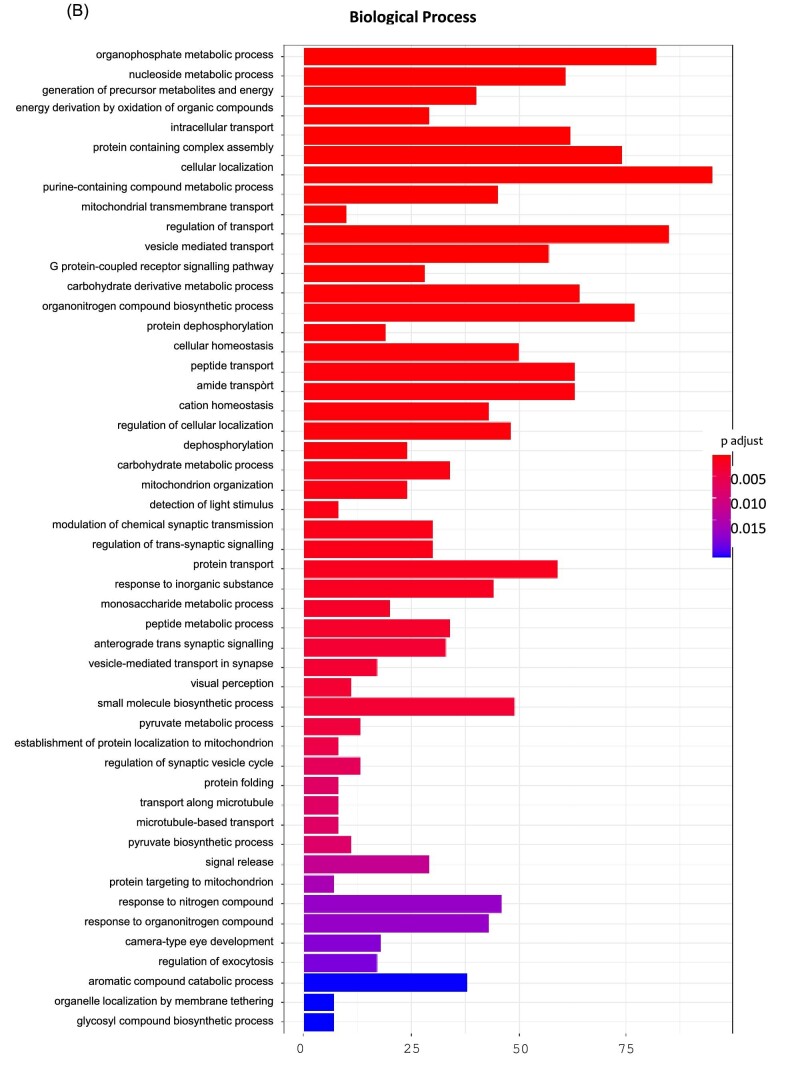
Network of biological processes where retinal proteins binding Zinc, Magnesium, Calcium, Selenium, and Manganese are involved.

Over-represented GO biological processes of retinal putative metal/metalloid-binding proteins include (Fig. 10): (1) generation of precursor metabolites and energy, (2) energy derivation by oxidation of organic compounds, (3) cellular respiration, (4) ATP metabolic processes, (5) protein-containing complex subunit organization, (6) regulation of transport, (7) nitrogen compound transport, and (8) organic substance transport. The biological processes clustering by specific metal/metalloid binding proteins can be seen in Supplementary Fig. 2.

Putative zinc-binding proteins were found to be in the following biological processes: (1) protein-containing complex assembly, (2) protein-containing complex subunit organization, (3) RNA processing, (4) tRNA aminoacylation for protein translation, (5) RNA processing, (6) translation initiation complex formation, (7) aminoacid activation, (8) cellular component biogenesis, (9) localization to the cell.

Putative magnesium-binding proteins were found to be involved in: (1) protein dephosphorylation, (2) organophosphate metabolic process, (3) nucleobase-containing small molecule metabolic process, (4) nucleoside phosphate metabolic process.

Putative calcium-binding proteins biological processes: (1) calcium ion homeostasis, (2) divalent inorganic cation homeostasis, (3) cell contraction, (4) glycerophospholipid biosynthesis, (5) cell conduction, (6) vesicle mediated transport, (7) localization to the cell.

Selenium-binding proteins biological processes: (1) cellular redox homeostasis, (2) hydrogen peroxide metabolic process.

Putative manganese-binding proteins biological processes: (1) pyruvate metabolism, (2) citric acid (TCA) cycle, (3) respiratory electron transport, (4) protein and amino acid modification, (5) protein dephosphorylation, (6) ribonucleotide processing, (7) generation of energy, (8) generation of precursor metabolites, (9) carbohydrate metabolism, (10) NADP metabolism, (11) isocitrate metabolism.

### Retinal proteins annotated as binding more than one metal

This group of proteins is very important as their biological processes could potentially be affected by the dyshomeostases of various metals directly and/or indirectly.

Biological processes of putative magnesium and manganese-binding proteins include: (1) ribose phosphate metabolic process, (2) nucleotide metabolic process, (3) protein dephosphorylation, (4) generation of precursor metabolites and energy, (5) dicarboxylic acid metabolic process, (6) peptidyl-threonine dephosphorylation. Putative magnesium and calcium-binding proteins are involved in the regulation of transport.

### Proteins involved in retina light processes and the visual cycle

Table 4 shows 21 retinal putative metal/metalloid-binding proteins involved in ‘detection of light stimulus’, ‘response to light stimulus ‘, ‘camera-type eye development’, ‘visual system development’, ‘visual learning’ (Fig. 9, Supplementary 4). The metal is annotated as having a role as a cofactor, allosteric modulator, structural component.

**Table 4.** Mouse (Mus musculus) putative neuroretina metal-binding proteins involved in ‘detection of light stimulus’, ‘response to light stimulus’, ‘camera-type eye development’, ‘visual system development’ (biological processes), molecular functions, localisation and retinal cell expression (A); length (amino acids), metal, metal liganding site (residues), domain (e.g. EF hand, Zinc fingers) and metal role (B). Amino acids symbols: Alanine (A), Arginine (R), Asparagine (N), Aspartic (D), Cysteine (C), Glutamine (Q), Glutamic acid (E), Glycine (G), Histidine (H), Isoleucine (I), Leucine (L), Lysine (K), Methionine (M), Phenylalanine (F), Proline (P), Serine (S), Threonine (T), Tryptophan (W), Tyrosine (Y), Valine (V)

Protein name Gene name	Molecular functions	Biological processes	Localisation Retinal cell expression	
(**A**)				
Rhodopsin Rho	G protein-coupled photoreceptor activity Vitamin binding 11-cis retinal binding Metal ion binding Retinal binding Spectrin binding	Detection of light stimulus Camera-type eye development Visual system development G protein-coupled receptor signalling pathway	Rod-shaped photoreceptor cells in the retina (at protein level) Rod photoreceptor outer segment Membrane Cell projection, cilium	
Recoverin Rcvrn	Calcium binding	Response to light stimulus Regulation of transport	Photoreceptor inner segment Cell projection, cilium, photoreceptor outer segment Photoreceptor outer segment membrane Perikaryon Note: Primarily expressed in the inner segments of light-adapted rod photoreceptors, approximately 10% of which translocates from photoreceptor outer segments upon light stimulation	
Guanine nucleotide-binding protein G(t) subunit alpha-1 Gnat1	G protein-coupled receptor signalling pathway GTPase activity	Response to light stimulus Detection of light stimulus Camera-type eye development Visual system development	Cell projection, cilium, photoreceptor outer segment Membrane Peripheral membrane protein Photoreceptor inner segment	
Guanine nucleotide-binding protein G(t) subunit alpha-2 (transducin) Gnat2	G-protein beta/gamma-subunit complex binding GTPase activity	Response to light stimulus Detection of light stimulus Camera-type eye development Visual system development G protein-coupled receptor signalling pathway Cellular homeostasis Cation homeostasis	Cell projection, cilium, photoreceptor outer segment Membrane Peripheral membrane protein Photoreceptor inner segment	
Guanylyl cyclase-activating protein 1 Guca1a	Calcium sensitive guanylate cyclase activator activity	Response to light stimulus Organophosphate metabolic process Nucleotide metabolic process purine-containing compound Metabolic process Organonitrogen compound biosynthetic process Regulation of small molecule metabolic process	Membrane Lipid-anchor Photoreceptor inner segment Cell projection, cilium photoreceptor outer segment	
Phospholipid-transporting ATPase IB Atp8a2	Magnesium ion binding Aminophospholipid flippase activity ATPase-coupled intramembrane lipid transporter activity ATP binding Phosphatidylethanolamine flippase activity Phosphatidylserine flippase activity	Response to light stimulus Visual system development Regulation of transport	Photoreceptor outer segment membrane Photoreceptor inner segment membrane Localizes to disk membranes of rod photoreceptor outer segments (ROS) Localizes to the Golgi and endosomes in photoreceptor cells Membrane Multi-pass membrane protein Golgi apparatus membrane Endosome membrane Cell membrane	
Rod cGMP-specific 3′,5′-cyclic phosphodiesterase subunit beta Pde6b	3′,5′-cyclic-GMP phosphodiesterase activity 3′,5′-cyclic-nucleotide phosphodiesterase activity metal ion binding	Response to light stimulus Detection of light stimulus Camera-type eye development Visual system development Cellular homeostasis G protein-coupled receptor signalling pathway Response to nitrogen compound Response to organonitrogen compound	Membrane Cell projection, cilium Photoreceptor outer segment	
Dual specificity calcium/calmodulin-dependent 3′,5′-cyclic nucleotide phosphodiesterase 1B Pde1b	Calmodulin binding calmodulin-dependent cyclic-nucleotide phosphodiesterase activity cyclic-nucleotide phosphodiesterase activity metal ion binding	Response to light stimulus Regulation of neurotransmitter levels	Cytoplasm, cytosol	
Calcium-binding protein 4 (CaBP4) Cabp4	Calcium channel regulator activity Calcium ion binding Ion channel binding	Response to light stimulus Phototransduction Visual perception Camera-type eye development Visual system development Photoreceptor cell morphogenesis Retinal bipolar neuron differentiation Retinal cone cell development	Cytoplasm Presynapse	
Mitochondrial adenyl nucleotide antiporter SLC25A25 Slc25a25	ATP transmembrane transporter activity Calcium ion binding	Camera-type eye development Visual system development Organophosphate metabolic process Nucleotide metabolic process Generation of precursor metabolites and energy Energy derivation by oxidation of organic compound Purine-containing compound metabolic process Carbohydrate derivative metabolic process	Mitochondrion inner membrane	
Peptidyl-prolyl cis-trans isomerase FKBP8 Fkbp8	Disordered domain specific binding Identical protein binding Peptidyl-prolyl cis-trans isomerase activity Protein folding chaperone	Camera-type eye development Visual system development	Mitochondrion membrane	
Calbindin Calb1	Cellular homeostasis Vitamin binding Calcium ion binding Involved in regulation of postsynaptic cytosolic calcium ion concentration Calcium ion binding involved in regulation of presynaptic cytosolic calcium ion concentration Vitamin D binding Zinc ion binding	Camera-type eye development Visual system development Retina layer formation Cation homeostasis Modulation of chemical synaptic transmission Anterograde trans-synaptic signaling	Horizontal cells Cones (both L/M and S cones) Axon Cytoplasm Cytosol Dendrite Dendritic spine GABA-ergic synapse Glutamatergic synapse Neuron projection Neuronal cell body Nucleus Postsynapse Postsynaptic cytosol Presynaptic cytosol Stereocilium synapse Terminal bouton	
Reticulocalbin-1 Rcn1		Camera-type eye development Visual system development	Endothelial cells Endoplasmic reticulum lumen	
Lysophosphatidylcholine acyltransferase 1 Lpcat1	1-acylglycerol-3-phosphate O-acyltransferase activity Acylglycerophosphocholine O-acyltransferase activity Alkenylglycerophosphocholine O-acyltransferase activity Alkylglycerophosphocholine O-acetyltransferase activity Alkylglycerophosphocholine O-acyltransferase activity Calcium ion binding Plasmalogen synthase activity	Camera-type eye development Visual system development Organophosphate metabolic process Organonitrogen compound biosynthetic process	Endoplasmic reticulum membrane Pass type II membrane protein Golgi apparatus membrane Single-pass type II membrane protein Lipid droplet	
Glycerol-3-phosphate dehydrogenase, mitochondrial Gpd2	Oxidoreductase activity, acting on the CH-OH group of donors, NAD or NADP as acceptor	Camera-type eye development Visual system development Organophosphate metabolic process Nucleotide metabolic process Carbohydrate derivative metabolic process Carbohydrate metabolic process Monosaccharide metabolic process Small molecule biosynthetic process	Mitochondrion inner membrane	
Integrin beta-1 Itgb1	Intracellular transport	Response to light stimulus Visual learning	Muller cells Cell membrane protrusions Localized at plasma and ruffle Cell membrane, sarcolemma Cell junction	
AMP-activated protein kinase catalytic subunit alpha-1 Prkaa1	protein C-terminus binding	Response to light stimulus (UV light) Organophosphate metabolic process nucleotide metabolic process Generation of precursor metabolites and energy Intracellular transport Protein-containing complex assembly Cellular localization Purine-containing compound metabolic process Regulation of transport Vesicle-mediated transport Carbohydrate derivative metabolic process Organonitrogen compound biosynthetic process Cellular homeostasis Peptide transport Amide transport Regulation of cellular localization Carbohydrate metabolic process Mitochondrion organization Protein transport Response to inorganic substance Monosaccharide metabolic process Small molecule biosynthetic process Pyruvate metabolic process Establishment of protein localization to mitochondrion Pyruvate biosynthetic process Protein targeting to mitochondrion Response to nitrogen compound Response to organonitrogen compound Aromatic compound catabolic process Regulation of small molecule metabolic process Cellular nitrogen compound catabolic process Heterocycle catabolic process Cellular response to hormone stimulus Regulation of organelle organization Ribonucleoprotein complex biogenesis	Cytoplasm Nucleus	
ATP-dependent DNA/RNA helicase DHX36 Dhx36	Magnesium ion binding Catalytic activity, acting on RNA	Response to light stimulus (UV light) Protein-containing complex assembly Cellular localization Regulation of transport Organonitrogen compound biosynthetic process Peptide transport Amide transport Regulation of cellular localization Protein transport Response to inorganic substance Peptide metabolic process Response to nitrogen compound Response to organonitrogen compound Response to organonitrogen compound Aromatic compound catabolic process RNA processing Synapse organization Cellular nitrogen compound catabolic process Heterocycle catabolic process Regulation of organelle organization	Nucleus Cytosol	
Sodium/potassium-transporting ATPase subunit alpha-2 Atp1a2	chaperone binding ATPase activity, coupled to transmembrane movement of ions, phosphorylative mechanism	Visual learning Organophosphate metabolic process Nucleotide metabolic process Purine-containing compound metabolic process Regulation of transport Carbohydrate derivative metabolic process Cellular homeostasis Cation homeostasis Regulation of neurotransmitter levels Circulatory system process Cellular response to hormone stimulus	Muller cells Membrane Multi-pass membrane protein Cell membrane	
Serine/threonine-protein phosphatase PP1-alpha catalytic subunit Ppp1ca	Phosphatase activity	Response to light stimulus (circadian cycle) Generation of precursor metabolites and energy Energy derivation by oxidation of organic compounds Organonitrogen compound biosynthetic process Protein dephosphorylation Dephosphorylation Carbohydrate metabolic process Response to inorganic substance Monosaccharide metabolic process Peptide metabolic process Regulation of small molecule metabolic process	Cytoplasm Nucleus Nucleus, nucleoplasm Nucleus, nucleolus	
Serine/threonine-protein phosphatase PP1-beta catalytic subunit Ppp1cb	Phosphatase activity	Response to light stimulus (circadian rhythm) Generation of precursor metabolites and energy Energy derivation by oxidation of organic compounds Protein dephosphorylation Dephosphorylation Carbohydrate metabolic process Monosaccharide metabolic process Regulation of small molecule metabolic process	Cytoplasm Nucleus Nucleus, nucleoplasm Nucleus, nucleolus	
(**B**)				
**Protein name(s)** **Gene name** **Length (aminoacids)**	**Metal**	**Metal liganding** **Position** **(Residue)**	**Domain/motifs** **Amino acids sequence**	**Metal role**
Rhodopsin Rho (348)	Zn^2+^	201 (E) 279 (Q)		Structural Cofactor Catalytic Allosteric modulator
Recoverin Rcvrn (202)	Ca^2+^ Ca^2+^ Ca^2+^ Ca^2+^ Ca^2+^ Ca^2+^ Ca^2+^ Ca^2+^ Ca^2+^ Ca^2+^	74 (D) 76 (N) 78 (D) 80 (T) 85 (E) 110 (D) 112 (D) 114 (N) 116 (T) 121 (E)	EF-hand 1, 41-59 SGRITRQEFESIYSKFFPD EF-hand 2, 61-96 DPKAYAQHVFRSFDANSDGTLDFKEYVIALHMTTAG EF-hand 3, 97-132 KPTQKLEWAFSLYDVDGNGTISKNEVLEIVMAIFKM EF-hand 4, 147-182 TPEKRAEKIWAFFGKKEDDKLTEEEFIEGTLANKEI	Cofactor
Guanine nucleotide-binding protein G(t) subunit alpha-1 Gnat1 (350)	Mg^2+^ Mg^2+^ Mg^2+^	43 (S) 177 (T)		Cofactor
Guanine nucleotide-binding protein G(t) subunit alpha-2 Gnat2 (354)	Mg^2+^ Mg^2+^	47 (S) 181 (T)		Cofactor
Guanylyl cyclase-activating protein 1 Guca1a (202)	Ca^2+^ Ca^2+^ Ca^2+^ Ca^2+^ Ca^2+^ Ca^2+^ Ca^2+^ Ca^2+^ Ca^2+^ Ca^2+^ Ca^2+^ Ca^2+^ Ca^2+^ Ca^2+^ Ca^2+^	64 (D) 66 (N) 68 (D) 70 (Y) 75 (E) 100 (D) 102 (D) 104 (N) 106 (C) 111 (E) 144 (D) 146 (D) 148 (D) 150 (E) 155 (E)	EF-hand 1, 31-49 SGQLTLYEFRQFFGLKNLS EF-hand 2, 51-86 SASQYVEQMFETFDFNKDGYIDFMEYVAALSLVLKG EF-hand 3 87-122 KVEQKLRWYFKLYDVDGNGCIDRDELLTIIRAIRTI EF-hand 4, 131-166 SAEEFTDTVFAKIDINGDGELSLEEFMEGVQKDQML	Structural
Phospholipid-transporting ATPase IB Atp8a2 (1148)	Mg^2+^ Mg^2+^ Mg^2+^ Mg^2+^	388 (D) 390 (T) 781 (D) 785 (D)		Cofactor
Rod cGMP-specific 3′,5′-cyclic phosphodiesterase subunit beta Pde6 (856)	Zin^2+^/Mg^2+^/Mn^2+^ Zin^2+^/Mg^2+^/Mn^2+^ Zin^2+^/Mg^2+^/Mn^2+^ Zin^2+^/Mg^2+^/Mn^2+^ Zin^2+^/Mg^2+^/Mn^2+^	561 (H) 597 (H) 598 (D) 598 (D) 718 (D)		Cofactor
Dual specificity calcium/calmodulin-dependent 3′,5′-cyclic nucleotide phosphodiesterase 1B Pde1b (535)	Zn^2+^ Zn^2+^ Zn^2+^/Mg^2+^ Zn^2+^	226 (H) 262 (H) 263 (D) 369 (D)		Cofactor
Calcium-binding protein 4 (CaBP4) Cabp4 (271)	Ca^2+^ Ca^2+^ Ca^2+^ Ca^2+^ Ca^2+^ Ca^2+^ Ca^2+^ Ca^2+^ Ca^2+^ Ca^2+^ Ca^2+^ Ca^2+^ Ca^2+^ Ca^2+^ Ca^2+^	138 (D) 140 (D) 142 (D) 144 (Y) 149 (E) 215 (D) 217 (D) 219 (D) 221 (R) 226 (E) 252 (D) 254 (N) 256 (D) 258 (T) 263 (E)	EF-hand 1, 125-160 EELEELQAAFEEFDTDQDGYIGYRELGDCMRTLGYM EF-hand 2, 179-196 GFVDFEEFVELISPKLRE EF-hand 3202-237 LGVRELRIAFREFDKDRDGRITVAELRQAAPALLGE EF-hand 4, 239-271 LEGTELDEMLREMDLNGDGTIDFDEFVMMLSTG	Structural
Mitochondrial adenyl nucleotide antiporter SLC25A25 Slc25a25 (469)	Ca^2+^ Ca^2+^ Ca^2+^ Ca^2+^ Ca^2+^	60 (D) 62 (D) 64 (D) 66 (Q) 71 (E)	EF-hand 1, 47-80 TYRQWKQKIVQAGDKDLDGQLDFEEFVHYLQDHE EF-hand 2, 78-113 DHEKKLRLVFKSLDKKNDGRIDAQEIMQSLRDLGVK EF-hand 3, 114-149 ISEQQAEKILKSMDKNGTMTIDWNEWRDYHLLHPVE	Structural
Peptidyl-prolyl cis-trans isomerase FKBP8 Fkbp8 (402) Ca^2+^ Cofactor	Ca^2^	N/A	N/A	Cofactor
Calbindin Calb1 (261)	Ca^2+^ Ca^2+^ Ca^2+^ Ca^2+^ Ca^2+^ Ca^2+^ Ca^2+^ Ca^2+^ Ca^2+^ Ca^2+^ Ca^2+^ Ca^2+^ Ca^2+^ Ca^2+^ Ca^2+^ Ca^2+^ Ca^2+^ Ca^2+^ Ca^2+^	24 (D) 26 (D) 28 (S) 30 (Y) 35 (E) 111 (D) 113 (D) 115 (S) 122 (E) 155 (D) 157 (N) 159 (D) 161 (K) 166 (E) 199 (D) 201 (D) 203 (N) 205 (Y) 210 (S)	EF-hand 1, 11-46 ITASQFFEIWLHFDADGSGYLEGKELQNLIQELLQA EF-hand 2, 53-88 ELSPEMKSFVDQYGQRDDGKIGIVELAHVLPTEENF EF-hand 3, 98-133 KSCEEFMKTWRKYDTDHSGFIETEELKNFLKDLLEK EF-hand 4, 142-177 KLAEYTDLMLKLFDSNNDGKLELTEMARLLPVQENF EF-hand 5, 186-221 MCGKEFNKAFELYDQDGNGYIDENELDALLKDLCEK	Structural
Reticulocalbin-1 Rcn1 (325)	Ca^2^ Ca^2^ Ca^2^ Ca^2^ Ca^2^ Ca^2^ Ca^2^ Ca^2^ Ca^2^ Ca^2^ Ca^2^ Ca^2^ Ca^2^ Ca^2^ Ca^2^ Ca^2^ Ca^2^ Ca^2^ Ca^2^ Ca^2^ Ca^2^ Ca^2^ Ca^2^ Ca^2^ Ca^2^ Ca^2^ Ca^2^ Ca^2^	86 (D) 88 (D) 90 (D) 97 (E) 122 (D) 124 (D) 126 (D) 128 (K) 133 (E) 173 (D) 175 (D) 177 (D) 179 (T) 184 (E) 210 (D) 212 (N) 214 (D) 221 (E) 251 (D) 253 (N) 255 (D) 257 (K) 262 (E) 287 (D) 289 (N) 291 (D) 293 (M) 298 (E)	EF-hand 1, 73-108 ESKERLGKIVDRIDSDGDGLVTTEELKLWIKRVQKRYIYDNVAKVW EF-hand 2, 109-144 YIYDNVAKVWKDYDRDKDEKISWEEYKQATYGYYLG EF-hand 3, 160-195 KMLPRDERRFKASDLDGDLTATREEFTAFLHPEEFE EF-hand 4, 197-232 MKEIVVLETLEDIDKNGDGFVDQDEYIADMFSHEDN EF-hand 5, 238-273 WVLSEREQFNDFRDLNKDGKLDKDEIRHWILPQDYD EF-hand 6, 274-309 HAQAEARHLVYESDKNKDEMLTKEEILDNWNMFVGS	Structural
Lysophosphatidylcholine acyltransferase 1 Lpcat1 (534)	Ca^2+^ Ca^2^ Ca^2^ Ca^2^	392 (D) 394 (S) 398 (E) 403 (E)	EF-hand 1, 379-414 PSEEEKRNPALYASNVRRVMAKALGVSVTDYTFEDCQLALAEGQLRLPADTCLLEFARLVRGLGLKPENLEKDLDKYSESARMKRGEKIRLPEFAAYLEVPVSDALEDMFSLFDESGGGEIDLREYVVALSVVCRP EF-hand 2, 451-486 VSELTVTDLFQAIDQEDKGRITFDDFCGFAEMYPDYA	Structural
Glycerol-3-phosphate dehydrogenase, mitochondrial Gpd2 (727)	Ca^2^ Ca^2^ Ca^2^ Ca^2^ Ca^2^	672 (D) 674 (N) 676 (N) 678 (Q) 683 (E)	EF-hand 1, 623-658 SDIDRYKKRFHKFDEDEKGFITIVDVQRVLESINVQ EF-hand 2, 659-694 MDENTLHEILCEVDLNKNGQVELHEFLQLMSAVQKG	Structural
Integrin beta-1 Itgb1 (798)	Mg^2+^ Ca^2+^/Mg^2+^ Ca^2^ Ca^2^ Ca^2^ Ca^2^ Ca^2^ Ca^2^ Ca^2^/Mg^2+^ Ca^2^	152 (S) 154 (S) 157 (D) 158 (D) 189 (E) 244 (N) 246 (D) 248 (P) 249 (E) 362 (G)		Cofactor
5'-AMP-activated protein kinase catalytic subunit alpha-1 (AMPK subunit alpha-1) Prkaa1 (559)	Mg^2+^	N/A		Cofactor
ATP-dependent DNA/RNA helicase DHX36 Dhx36 (1001)	Mg^2+^ Mg^2+^	328 (E) 330 (H)		Cofactor
Sodium/potassium-transporting ATPase subunit alpha-2 Atp1a2 (1020)	Mg^2+^ Mg^2+^	714 (D) 718 (D)		Cofactor
Serine/threonine-protein phosphatase PP1-alpha catalytic subunit Ppp1ca (330)	Mn^2+^ Mn^2+^ Mn^2+^ Mn^2+^ Mn^2+^ Mn^2+^ Mn^2+^	64 (D) 66 (H) 92 (D) 92 (D) 124 (N) 173 (H) 248 (H)		Cofactor
Serine/threonine-protein phosphatase PP1-beta catalytic subunit Ppp1cb (327)				Cofactor

They include 9 calcium-binding proteins, 6 magnesium-binding, 2 manganese-binding, and 1 zinc-binding. Three proteins bind more than one metal.

Calcium-binding proteins: (1) recoverin, (2) GCAP1, (3) calmodulin phosphodiesterase 1B, (4) calcium-binding protein 4 (CaBP4), (5) calcium-binding mitochondrial carrier protein SMaMC-2, (6) rotamase, (7) reticulocalbin 1, (8) lysophosphatidylcholine acyltransferase 1, (9) mitochondrial glycerol 3-phosphate dehydrogenase.

Putative magnesium-binding proteins: (1) transducin subunit alpha 1, (2) transducin subunit alpha 2, (3) ATPase 1B, (4) AMP protein kinase subunit alpha 1, (5) ATP dependent DNA/RNA helicase DHX36, (6) Na/K ATPase subunit alpha 2.

Putative manganese-binding proteins: (1) serine/threonine protein phosphatase PP1 alpha catalytic subunit, (2) serine/threonine protein phosphatase PP1 beta catalytic subunit.

Zinc-binding protein: rhodopsin.

Rod GMP phosphodiesterase beta binds zinc, manganese and magnesium. Fibronectin receptor subunit beta (integrin beta 1) binds magnesium and calcium. Calbindin D28 binds calcium and zinc.

A list of 13 visual cycle proteins was obtained from Tsin *et al*.^29^ The PRIDE dataset was filtered for visual cycle proteins with 8 of the 13 being found in all 3 datasets (PXD009909, PXD009981, and PXD003441) (Table 5). The overlap of visual cycle proteins is shown in the Venn diagram (Supplementary Fig. 1), showing only 1 protein was not found in any proteomic dataset. All protein information for visual cycle proteins can be found in the Supplementary 6.

**Table 5.** Putative metal binding proteins found in the retinal pigment epithelium (RPE) microvilli. Mouse (Mus musculus**).** A) Biological processes, molecular functions, localisation and expression. (B) length (amino acids), metal, metal liganding site (residues), domain (e.g. EF hand, Zinc fingers) and metal role. (B) length (amino acids), metal, metal liganding site (residues), domain (e.g. EF hand, Zinc fingers) and metal role (B)**.**Amino acids symbols: Alanine (A), Arginine (R), Asparagine (N), Aspartic (D), Cysteine (C), Glutamine (Q), Glutamic acid (E), Glycine (G), Histidine (H), Isoleucine (I), Leucine (L), Lysine (K), Methionine (M), Phenylalanine (F), Proline (P), Serine (S), Threonine (T), Tryptophan (W), Tyrosine (Y), Valine (V)

Protein name Gene name	Molecular functions	Biological processes	Localisation Expression	
**A**) Alpha-enolase Eno1	Lyase activity DNA-binding transcription repressor activity RNA polymerase II-specific enzyme binding GTPase binding Heat shock protein binding Identical protein binding Magnesium ion binding Phosphopyruvate hydratase activity Protein-containing complex binding Protein homodimerization activity RNA binding RNA polymerase II transcription regulatory D region sequence-specific DNA binding	Regulation of vacuole fusion, non-autophagic Canonical glycolysis Cellular response to interleukin-7 Glycolytic process In utero embryonic development Positive regulation of binding Organophosphate metabolic process	Cytoplasm Cell membrane	
Gamma-enolase Eno2	Enzyme binding Identical protein binding Magnesium ion binding Phosphopyruvate hydratase activity Protein-containing complex binding Heat shock protein binding	Regulation of vacuole fusion, non-autophagic Nucleotide metabolic process Glycolytic process	Cytoplasm Cell membrane	
Carbonic anhydrase 14 Cah14	Carbonate dehydratase activity Hydro-lyase activity Zinc ion binding	Carbon dioxide transport One-carbon metabolic process Regulation of pH	Membrane	
Sodium/potassium-transporting ATPase subunit alpha-1 (Na(+)/K(+) ATPase alpha-1 subunit) At1a1	Translocase ADP binding Ankyrin binding ATP binding Chaperone Phosphatase activity Phosphatidylinositol 3-kinase binding Potassium ion binding Protein domain specific binding Protein heterodimerization activity Protein kinase binding Sodium:potassium-exchanging ATPase activity Sodium ion binding Steroid hormone binding	Na,K-transporting ATPase α1 chain	Cell membrane Basolateral cell membrane Sarcolemma Cell projection Melanosome	
Pyruvate kinase PKM Pkm	ADP binding ATP binding Identical protein binding Kinase activity Magnesium ion binding	Pyruvate kinase, M2 isozyme	Cytoplasm Nucleus	
Integrin alpha-V Itav	C-X3-C chemokine binding Extracellular matrix binding Extracellular matrix protein binding Fibroblast growth factor binding Fibronectin binding Insulin-like growth factor I binding Integrin binding Metal ion binding Neuregulin binding Protease binding Signaling receptor binding Transforming growth factor beta binding Voltage-gated calcium channel activity	Vitronectin receptor α subunit (integrin αv)	Cell membrane Cell junction, focal adhesion	
**B**)				
**Protein name(s)** **Gene name** **Length (amino acids)**	**Metal**	**Metal liganding** **Position** **(Residue)**	**Domain** **Amino acids sequence**	**Metal role**
Alpha-enolase Eno1 (434)	Mg^2+^ Mg^2+^ Mg^2+^ Mg^2+^	40 (S) 245 (D) 293 (E) 318 (D)	N/A	Cofactor
Gamma-enolase Eno2 (434)	Mg^2+^ Mg^2+^ Mg^2+^ Mg^2+^	40 (S) 245 (D) 293 (E) 318 (D)	N/A	Cofactor
Carbonic anhydrase 14 Cah14 (337)	Zn^2+^ Zn^2+^ Zn^2+^	109 (H) 111 (H) 135 (H)	N/A	Cofactor Catalysis
Sodium/potassium-transporting ATPase subunit alpha-1 (Na(+)/K(+) ATPase alpha-1 subunit) At1a1 (1023)	Mg^2+^ Mg^2+^	717 (D) 721 (D)	N/A	Cofactor
Pyruvate kinase PKM Pkm (531)	Mg^2+^ Mg^2+^	272 (E) 296 (D)	N/A	Cofactor
Integrin alpha-V Itav (1044)	Ca^2+^ Ca^2+^ Ca^2+^ Ca^2+^ Ca^2+^ Ca^2+^ Ca^2+^ Ca^2+^ Ca^2+^ Ca^2+^ Ca^2+^ Ca^2+^ Ca^2+^ Ca^2+^ Ca^2+^ Ca^2+^ Ca^2+^ Ca^2+^ Ca^2+^ Ca^2+^	260 (Y) 262 (L) 264 (Y) 266 (V) 268 (V) 314 (D) 316 (N) 318 (D) 320 (Y) 322 (D) 379 (D) 381 (D) 383 (D) 385 (D) 387 (D) 443 (D) 445 (D) 447 (N) 449 (Y) 451 (D)	N/A	Cofactor

Alpha-enolase, gamma-enolase, Na/K ATPase subunit alpha 1, and pyruvate kinase muscle isoenzyme annotated as binding magnesium. Integrin alpha V binding calcium. Carbonic anhydrase XIV binding zinc.

### Retinal putative metal/metalloid-binding proteins clinical variants associated with retinal disease

Table 6 shows clinical variants in eleven retinal putative metal/metalloid-binding proteins associated with retinal disease. These include rhodopsin, recoverin, guanylyl cyclase activating protein 1, transducin alpha 1 subunit, transducin alpha 2 subunit, ATPase 8A2, cGMP phosphodiesterase beta subunit, phosphodiesterase 1 beta, calcium binding protein 4, photoreceptor specific nuclear receptor.

**Table 6.** Human mutations in putative retinal metal binding proteins associated with retinal disease. Some mutations affecting the metal binding site or its proximity (i.e. Rhodopsin, Calcium binding protein 4, photoreceptor specific nuclear receptor). Amino acids symbols: Alanine (A), Arginine (R), Asparagine (N), Aspartic (D), Cysteine (C), Glutamine (Q), Glutamic acid (E), Glycine (G), Histidine (H), Isoleucine (I), Leucine (L), Lysine (K), Methionine (M), Phenylalanine (F), Proline (P), Serine (S), Threonine (T), Tryptophan (W), Tyrosine (Y), Valine (V). ad, autosomal dominant; ar, autosomal recessive; ESCS, enhanced S-cone syndrome.

Protein name Gene	Retina disease	Clinical Variant Human mutations Location, residues (pathological or likely pathological)
Rhodopsin Rho	• Leber's congenital amaurosis • Congenital night blindness • Retinitis pigmentosa (RP)	H211P ad retinitis pigmentosa 4
Calcium-binding protein 4 (CaBP4) Cabp4	• CABP4 retinopathy • Incomplete congenital stationary night blindness • Inner retinal dysfunction • Cone-rod synaptic transmission disorder with electronegative negative ERG • Foveal thinning • Normal autofluorescence • Cone-rod synaptic disorder, congenital nonprogressive • Cone dystrophy • Retinal dystrophy	R152Q, R47Q R225*, R120* N258I, N153I V158I, V263I R120Q, R225Q R253*, R148* L248P, L143P E48fs, E153fs R145C, R40C A229V, A124V R>* 225
Photoreceptor specific nuclear receptor Nr2e3	• Enhanced S-Cone Sensitivity Syndrome (ESCS) • Goldmann-Favre Syndrome • Clumped Pigmentary Retinal Degeneration • Retinitis Pigmentosa (RP)	R48C R48fs R48H V49L C50Y G56R ad retinitis pigmentosa K57R H58Q C64F C67Y G88V ar ESCS R97C R97H Q99L Q101* A102S A102D C103* R104Q ar ESCS R104W ar ESCS
Recoverin Rcvrn	• Autoantigen in paraneoplastic and degenerative diseases of the retina • Cancer-associated retinopathy	N/A
Guanine nucleotide-binding protein G(t) subunit alpha-1 Gnat1	• Autosomal dominant congenital stationary night blindness.	N/A
Guanine nucleotide-binding protein G(t) subunit alpha-2 Gnat2	• Autosomal dominant congenital stationary night blindness?	N/A
Guanylyl cyclase-activating protein 1 Guca1a	• Cone dystrophy 3 • Cone-rod dystrophy 14 • Dominant	N/A
Phospholipid-transporting ATPase IB Atp8a2	• Cerebellar ataxia, mental retardation, and disequilibrium syndrome (CAMRQ) and optic atrophy in humans • Motor neuron degeneration in mice • Recessive mutations progressive optic atrophy	N/A
Rod cGMP-specific 3′,5′-cyclic phosphodiesterase subunit beta Pde6b	• Retinitis pigmentosa • Autosomal dominant congenital stationary night blindness • Achromatopsia • Autosomal recessive RP in humans (rods). • Progressive retinal atrophy in dogs • Retinal degeneration in mice • Photoreceptor loss in mice	N/A
Dual specificity calcium/calmodulin-dependent 3′,5′-cyclic nucleotide phosphodiesterase 1B Pde1b	• ?	N/A

It is interesting to see there are three cases where the mutations affect the metal binding site or its proximity: mutations in rhodopsin, calcium binding protein 4 and photoreceptor specific nuclear receptor.

## Discussion

This study provides a comprehensive understanding of the overall metal/metalloid-related biological processes occurring in the retina, and identification of retinal putative metal/metalloid-binding proteins.

Metal/metalloid-binding proteins include metalloproteins (i.e. proteins that require the metal ion to perform their physiological function), and proteins involved in metal/metalloid transport, delivery, chaperones, storage, detoxification, and/or efflux.

The proportion of putative proteins binding specific metals/metalloid in the mouse retina seems to differ from the whole animal. We identified a higher proportion of proteins annotated as binding magnesium, calcium and selenium, and a lower proportion of proteins as binding zinc.

Magnesium is the most abundant free divalent cation and the second most abundant intracellular cation.^36,37^ It is mainly distributed in the cytosol and subcellular organelles. In the retina, magnesium has been shown to be particularly abundant in photoreceptors outer and inner segments.^38–40^ Magnesium ions bind proteins only weakly, which allows cells to switch enzymatic activity, which require magnesium for their catalytic action, on and off by changes in the local concentration of magnesium.

Magnesium deficiency has been associated with pigmentary retinal degeneration,^41^ retinitis pigmentosa,^42^ development or progression of diabetic retinopathy, retinal and choroidal calcification, myopia, as well as chorioretinitis.^43^^–^^45^ Retinal damage on a cellular scale has been associated with hypomagnesaemia in rats deprived of dietary magnesium in a scientific study in 2001.^38^ Magnesium deficiency resulted in multifocal necrosis in the RPE, deformation of surrounding photoreceptor OS, pyknosis of photoreceptor cell nuclei, and lamellar body inclusions in surviving RPE cells.

Over-represented GO molecular functions of retinal putative metal/metalloid-binding proteins found binding to diverse molecules, such as coenzyme, iron sulphur cluster, protein C terminus, protein beta/gamma subunit, calcium-dependent phospholipid, heat shock protein, chaperone, vitamin, NAD, amino acid, monosaccharide, GTPase activating protein, G-protein coupled, serotonin, AMP, quinone, protein phosphatase 2B, pyridoxal phosphate.

Over-represented enzyme activities among putative metal/metalloid binding retinal proteins included phosphatase, catalysis on RNA, lyase, metallopeptidase, ligase, GTPase, ribonuclease, electron transfer, oxidoreductase, ATPase, NADH dehydrogenase, phosphotransferase, aminoacyl-tRNA editing, metallochaperone, nucleoside-diphosphatase, phosphofructokinase, MAP kinase.

Cellular anatomical entities, where retinal putative metal/metalloid-binding proteins carry out their molecular function include: (1) mitochondrion (envelope, membrane, protein complex), (2) myelin sheath, (3) plasma membrane cell projections, and (4) neuron synapse projections.

As far as over-represented GO biological processes of retinal putative metal/metalloid-binding proteins, our analysis identified: (1) cellular localization, (2) ion homeostasis, (3) regulation of transport (e.g. nitrogen compounds, organic substances), (4) membrane potential, (5) RNA processing, (6) generation of precursor metabolites and energy, (7) energy derivation by oxidation of organic compounds, (8) cellular respiration, (9) ATP metabolic processes, (10) protein-containing complex subunit organization.

Manganese has various oxidation states and can function as a Lewis acid (like magnesium, zinc, calcium) or/and oxidation catalyst (like copper, iron, cobalt).^39^ Manganese has very similar chemical and complexation properties to magnesium and can replace it as ‘activating ion’ for a number of magnesium-dependent enzymes.^40^ In a biological context when replacing magnesium, the main oxidation state for Manganese would be 2+.

Putative calcium-binding protein’s biological processes identified include: (1) calcium ion homeostasis, (2) divalent inorganic cation homeostasis, (3) glycerophospholipid biosynthesis, (4) cell conduction, (5) vesicle mediated transport, and (6) localization to the cell. Retinal proteins annotated as binding both magnesium and calcium are involved in regulation of transport.

Putative zinc-binding protein’s biological processes identified: (1) protein-containing complex assembly, (2) protein-containing complex subunit organization, (3) RNA processing, (4) tRNA aminoacylation for protein translation, (5) RNA processing, (6) translation initiation complex formation, (7) aminoacid activation, (8) cellular component biogenesis, (9) localization to the cell.

Selenium-containing proteins have been divided into three groups: (1) specific selenium-binding proteins, (2) proteins into which selenium is incorporated non-specifically, and (3) proteins that contain selenium in the form of genetically encoded selenocysteine—selenoproteins.^46–48^ Selenocysteine is structurally similar to cysteine, except that the selenium atom in selenocysteine is present in place of the sulphur atom in cysteine. Selenocysteine has lower redox potential and higher catalytic activity than cysteine. At physiological pH, selenocysteine is almost completely ionized, while cysteine is protonated.

Identified mouse retina selenoproteins include oxidoreductases, antioxidants such as thioredoxin reductase, glutathione peroxidase and methionine sulfoxide reductase. Selenium-binding proteins biological processes include: (1) cellular redox homeostasis, (2) hydrogen peroxide metabolic process.

Twenty-one putative metal/metalloid-binding proteins were identified as involved in response to light stimulus and/or visual system development. We identified 11 proteins, where pathological or likely pathological clinical variants have been reported. ‘Disease associated’ with putative metal ion binding proteins does not seem to be high. Metal ion binding proteins per se might not be extraordinarily associated with disease. However, when disease-related mutations are identified in a putative metal-ion binding protein, a significant proportion of disease-related mutations are located in close proximity to the metal-ion binding site (i.e. rhodopsin, photoreceptor specific nuclear receptor, CaBP4). Changes in the amino acid residues contributing to the metal coordination site could result in alterations in the binding of the metal, protein structure, and/or function and associated retinal disease.

Rhodopsin is a G protein-coupled receptor expressed in photoreceptor OS with seven α-helices spanning the membrane. It is formed by a complex of a rod-type opsin with the chromophore ligand 11-cis-retinal. The opsin in humans is composed of 348 amino acid residues. Rhodopsin is the primary photoreceptor molecule of vision. It is required for phototransduction and photoreceptor viability after birth. Light induces isomerisation of the chromophore 11 cis-retinal to all-trans-retinal. This triggers a conformational change that activates the signalling via G-protein. Subsequent receptor phosphorylation mediates displacement of the bound G-protein alpha subunit by arrestin and terminates signalling.^49^

Zinc plays pivotal biochemical and structural roles in rhodopsin. Through examination of the rhodopsin crystal structure (PDB ID: 1L9H), Gleim *et al.*^50^ identified 4 putative Zn^2+^ coordination sites in each monomer of rhodopsin. These coordination sites have different roles: catalytic, cocatalytic, structural, affecting protein monomer interaction. The coordination residues are conserved (e.g.

human YYTLKPEVNNESFVIYMFVVHFTIPMIIIFFCYGQLVFTVK

mouse YYTLKPEVNNESFVIYMFVVHFTIPMIVIFFCYGQLVFTVK).

Putative amino acid residues in rhodopsin/opsin binding Zn^2+^. Interacting residues involved are usually in close 3D space.

Zn^2+^ 1 (histidine position 195 and glutamic acid 197), Zn^2+^ 2 (glutamic acid position 201 and glycine 279), Zn^2+^ 3 (histidine 211 and glutamic acid 122), Zn^2+^ 4 (Histidine position 100 of one monomer and lysine 311, arginine 314, and asparagine 315 of the second monomer).

The Zn^2+^ binding site in the transmembrane region is structurally important. Zn^2+^ here is coordinated by the side chains of two highly conserved residues, glutamic acid (position 122) in transmembrane helix III and histidine (position 211) in transmembrane helix V. Mutations in coordinating residues of this site reduce affinity for Zn^2+^. They can cause alterations in rhodopsin spectral properties, the structure of 11-cis-retinal binding pocket, rhodopsin thermal and dark stability, defects observed in retinitis pigmentosa. Residue change in position 211 from histidine to proline results in autosomal dominant Retinitis Pigmentosa 4 (https://www.ncbi.nlm.nih.gov/snp/rs28933993).

CaBP4 is composed of 275 amino acid residues in humans and 271 in mouse. It contains two separate domains (N and P) comprising four EF hands in two separate lobes. EF1 (residues 120–156) and EF2 (residues 163–190) form the N-lobe. EF3 (residues 200–230) and EF4 (residues 241–271) form the C-lobe. Ca^2+^ binds to EF1, EF3, and EF4. Mg^2+^ binds to EF1 and EF3. CaBP4 is involved in normal synaptic function through regulation of Ca^2+^ influx and neurotransmitter release in photoreceptor synaptic terminals.^51^

Pathogenic variants (cone-rod synaptic disorder, congenital nonprogressive, cone dystrophy, retinal dystrophy, ocular albinism, type II, cone-rod dystrophy, achromatopsia) have been reported with protein sequence variations in: (A) position 153 (glutamic acid missing), (B) position 219 (substitution of aspartic acid for histidine), (C) position 225 (arginine > *), (D) position 258 (asparagine substituted by isoleucine). (https://www.ncbi.nlm.nih.gov/snp/rs121917828, https://www.uniprot.org/uniprotkb/P57796/entry)

The photoreceptor-specific nuclear receptor (gene Nr2e3) is a ligand modulated transcriptional factor composed of 395 amino acid residues in mouse and 410 in human.^52^ It has a conserved structural organization consisting of several regions: (A) the A/B, in the N-terminus, a ligand-independent activator function (AF-1); (B) DNA-binding; (C) P,-box, thought to allow the receptor to bind to unique DNA response element sites and regulate gene transcription; (D) D-box, proposed to be involved in protein—protein interactions; (E) hinge domain linking the DNA- and ligand-binding domains; and (F) ligand-binding domain, in the C-terminal. The ligand of the photoreceptor-specific nuclear receptor is still unknown (i.e. orphan). The DNA-binding domain, which is very conserved, contains two cysteine-4-type zinc fingers. (https://www.uniprot.org/uniprotkb/Q9Y5×4/entry)

Mouse (*Mus musculus*)

ZnF1 position 40-60, amino acid sequence CRVCGDSSSGKHYGIYACNGC

ZnF2 position 76-101, CQVGAGMCPVDKAHRNQCQACRLKKC

Human (*Homo sapiens*)

ZnF1 position 47-67, amino acid sequence CRVCGDSSSGKHYGIYACNGC

ZnF2 position 83-108, CQVGAGMCPVDKAHRNQCQACRLKKC

The photoreceptor-specific nuclear receptor forms part of a complex network of signals that determine the fate of photoreceptor cell precursors (activator of rod development, repressor of cone development) and maintains the retina in adults.^53^ Rods contain a single type of visual pigment, rhodopsin, for high-sensitivity low-light vision. In contrast, human cones contain one of three alternative pigments (S-, M-, and L-opsins) each, which respond to short (S), medium (M), and long (L) wavelengths (i.e. blue, green, red, respectively) for colour and bright-light high-resolution vision. The photoreceptor specific nuclear receptor suppresses the expression of cone-specific genes (e.g. blue opsin, cone transducin subunits). It activates the expression of rod specific genes (e.g. rhodopsin, rod transducin subunits).^52,53^

More than 80 disease-causing variants of the photoreceptor specific nuclear receptor have been identified https://www.uniprot.org/uniprotkb/Q9Y5×4/variant; missense variant mutation in position 56 (glycine substituted by arginine) in the first zinc finger of the DNA-binding domain, causes autosomal dominant retinitis pigmentosa.^54–56^ This genotype—phenotype association has been found in 1–2% of autosomal dominant RP in North America, 3.5% in a large Spanish cohort [ref] and a large Belgian family. Substitution of glycine to valine at position 88, in zinc finger 2, results in autosomal recessive Enhanced S cone syndrome. SGFFKRSVRRRLIYRCQVGAGMCPVDKAHRNQCQACRLKKC, results in ESCS. These sequence variations may affect Zn^2+^ binding, protein structure, DNA-binding and transactivation function. Based on segregation analysis and bioinformatics predictions, Coppieters *et al.*^54^ hypothesized that this autosomal dominant causing mutation, p.G56R, may predominantly influence the terminal differentiation and maintenance of rods possibly through loss of interactions with zinc.

One of the limitations of this study, is the way UniProt identifies the putative metal-specific amino acid residues coordination, Annotations in Uniprot originate from curators, after identification from the literature, known 3D structures from PDB or inferred by sequence homology. The PDB currently contains more than 110 000 protein structures with approximately one third of which contain metal ions. In UniProt, at present, around 17% of curated proteins have annotated metal binding site residues. In the uncurated section, which contains the large majority of known protein sequences, just 3% of proteins have an annotated metal binding site. These annotations are created by a variety of automated annotation methods currently used. The difference in coverage between the reviewed (Swiss-Prot) and unreviewed (TrEMBL) suggests that there are many millions of missing putative metal binding site annotations in the 225 million TrEMBL sequences.

Further research is necessary to improve prediction of metal binding sites on proteins, identify the relationships between putative metal/metalloid-binding proteins specialised molecular functions, biological processes and potential benefits of interventions targeting metal homeostasis in the retina.

The use of the database MetalPDB (https://metalpdb.cerm.unifi.it) in future studies will be invaluable to expand our knowledge on the roles of metals and metalloids in retina physiology and disease. MetalPDB studies the metal binding sites and minimal functional sites of metal-binding proteins on the 3-D structure using structural information available in PDB.^57,58^

## Supplementary Material

mfae045_Supplemental_Files

## Data Availability

The data underlying this article are available from sources in the public domain, Uniport (https://www.uniprot.org/help/sequence_annotation) and ClinVar (http://www.ncbi.nlm.nih.gov/clinvar). UniProt is a freely accessible collection of databases with information on proteins sequences and functions. ClinVar^33^ is a freely accessible, public repository of genomic variations and their relationships to human phenotypes.
