# Supplementary figures and images for: Putative retina metal/metalloid-binding proteins: molecular functions, biological processes and retina disease associations
Source: Metallomics. 2024 Sep 25;16(10):mfae045. doi: 10.1093/mtomcs/mfae045 (PMC11523097; doi:10.1093/mtomcs/mfae045)

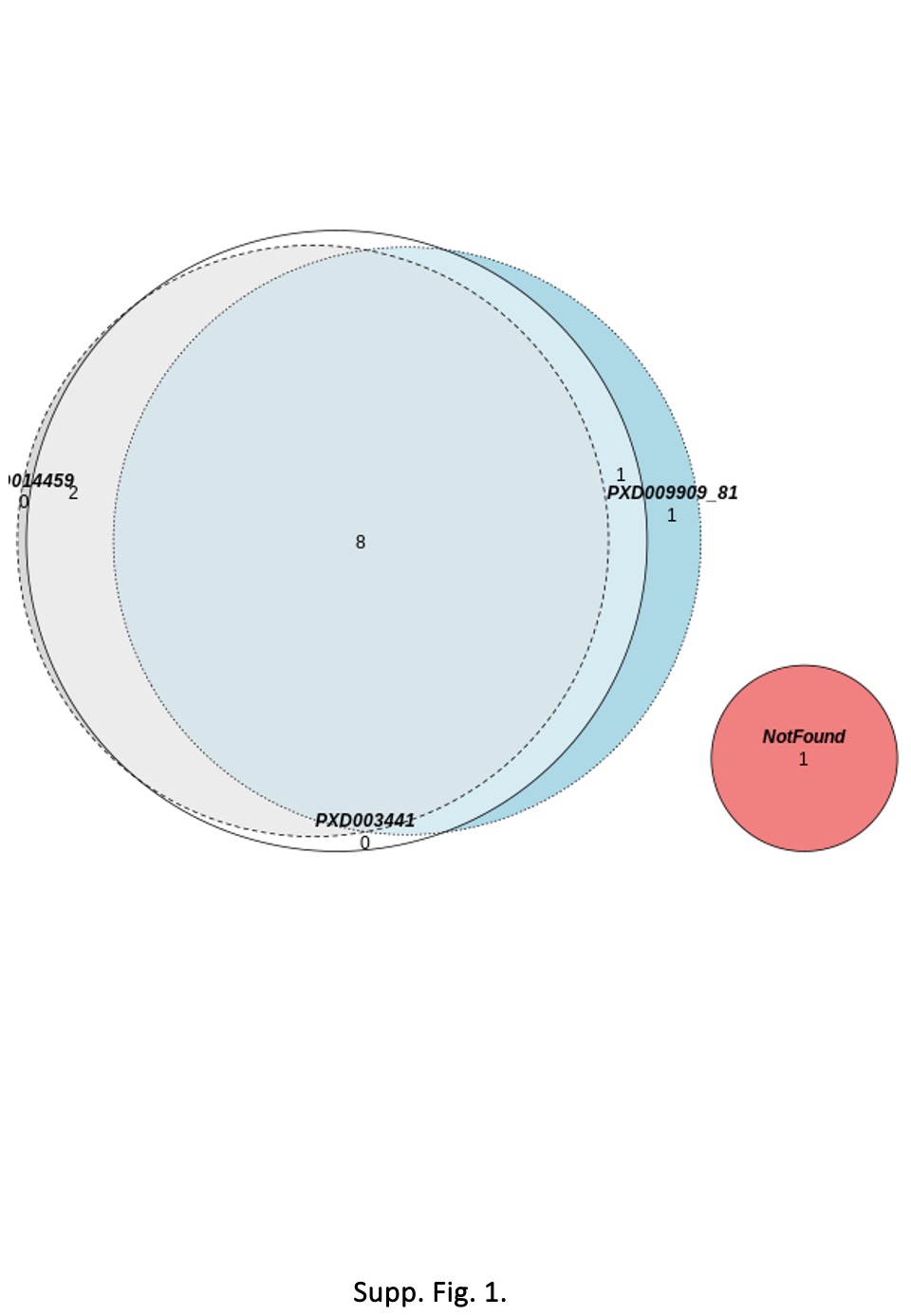

Supplement: mfae045_Supplemental_Files [file mfae045_supplemental_files.zip › Suppl_data_Supp_Fig_1.jpg]

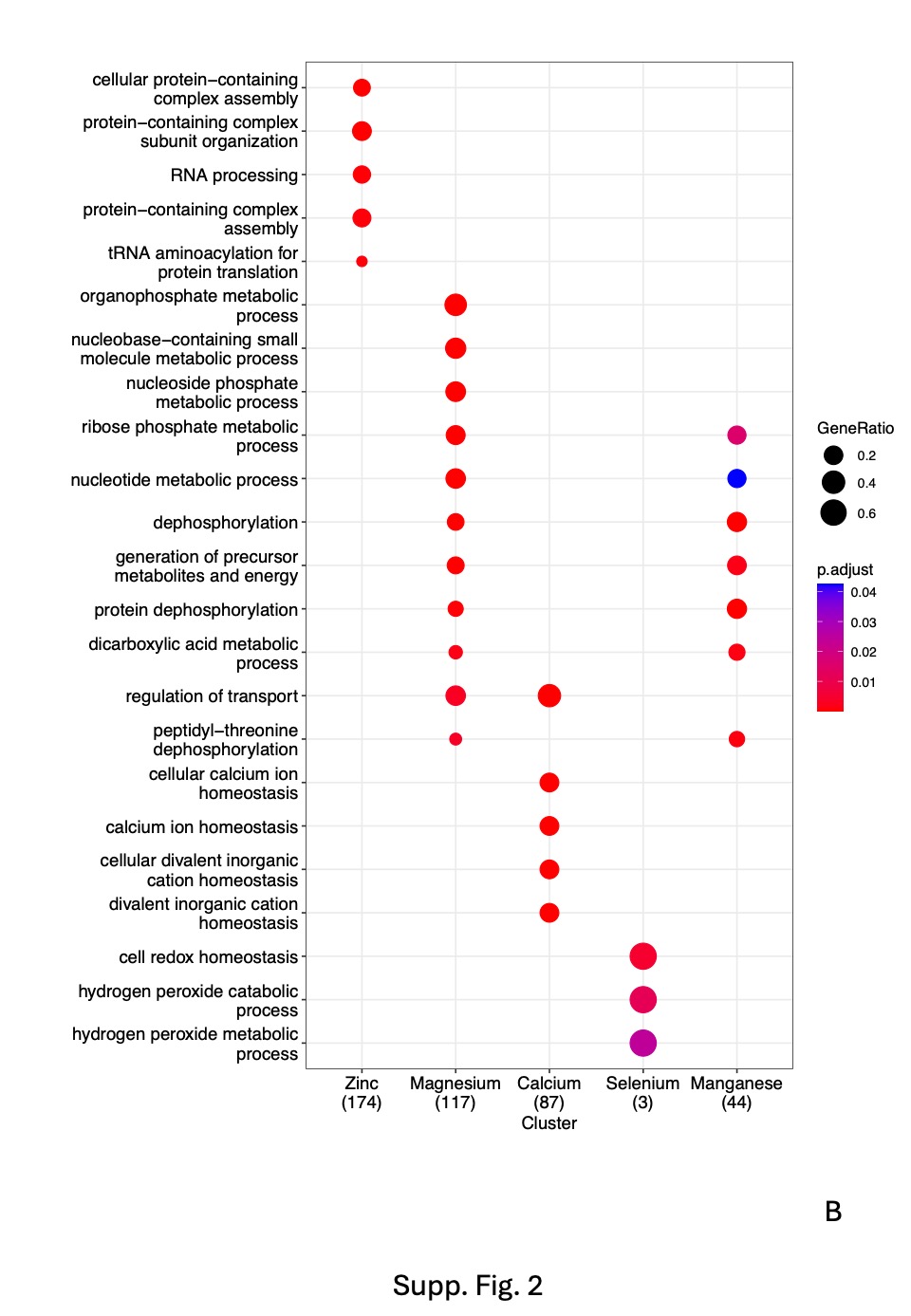

Supplement: mfae045_Supplemental_Files [file mfae045_supplemental_files.zip › Suppl_data_Supp_Fig_2.jpg]
